# Investigation of Complement Activation and Cytokine Signatures in AQP4+ NMOSD, MOGAD, and MS

**DOI:** 10.1002/acn3.70549

**Published:** 2026-10-21

**Authors:** Angeliki G. Filippatou, Susana Dominguez‐Penuela, David Acero‐Garces, Georgios Gakis, Matthew D. Smith, Yasmin Resto, Maria Reyes‐Mantilla, Haiwen Chen, Isabella Hernandez, Yana Said, Andrea Salazar‐Camelo, Christina Huynh, Alessia Tassoni, Ted Yednock, Rick Artis, Yaisa Andrews‐Zwilling, Scott D. Newsome, Shiv Saidha, Pavan Bhargava, Ellen M. Mowry, Kathryn C. Fitzgerald, Peter A. Calabresi, Carlos A. Pardo, Elias S. Sotirchos

**Affiliations:** ^1^ Department of Neurology Johns Hopkins University School of Medicine Baltimore Maryland USA; ^2^ Annexon Biosciences Brisbane California USA; ^3^ Department of Neurological Sciences University of Vermont School of Medicine Burlington Vermont USA; ^4^ Department of Pathology Johns Hopkins University School of Medicine Baltimore Maryland USA

**Keywords:** complement, cytokines, mogad, multiple sclerosis, neuromyelitis optica

## Abstract

**Objective:**

Aquaporin‐4‐IgG seropositive neuromyelitis optica spectrum disorder (AQP4+ NMOSD), myelin oligodendrocyte glycoprotein antibody‐associated disease (MOGAD), and multiple sclerosis (MS) are pathophysiologically distinct neuroinflammatory disorders. Complement activation is a major mechanism of injury in AQP4+ NMOSD, whereas its role in MS is complex and in MOGAD controversial. We aimed to compare complement components and cytokines between AQP4+ NMOSD, MOGAD, and MS.

**Methods:**

CSF complement components, CSF and plasma cytokines, neurofilament light chain (NfL) and glial fibrillary acidic protein (GFAP) were measured in people with AQP4+ NMOSD (CSF: *n* = 19; plasma: *n* = 18), MOGAD (CSF: *n* = 29; plasma: *n* = 35) and MS (CSF: *n* = 36; plasma: *n* = 58) with recent disease activity. Controls included subjects with non‐inflammatory conditions (CSF: *n* = 51) and healthy controls (plasma: *n* = 29).

**Results:**

CSF IL‐10 and TNF‐a were elevated across all neuroinflammatory disease groups relative to controls. CSF IL‐6 was elevated only in AQP4+ NMOSD and MOGAD. There were no significant differences in plasma cytokines between groups. Most complement components were elevated in AQP4+ NMOSD and MOGAD; only specific activation products (C2b, C3d, C4a, C5a) were elevated in MS. CSF GFAP and NfL correlated with several CSF cytokines and complement components in AQP4+ NMOSD and MOGAD. Expanded Disability Status Scale at nadir during attacks correlated with CSF BAFF, GFAP, free C1q, C1s, C1s‐C1inh, C4a in AQP4+ NMOSD, and CSF IL‐10, TNF‐a, free C1q, C1inh, C4a in MOGAD.

**Interpretation:**

Our findings suggest distinct CSF cytokine profiles in AQP4+ NMOSD, MOGAD, and MS, and widespread activation of complement pathways in AQP4+ NMOSD and MOGAD. CSF complement and cytokine profiling may help characterize neuroinflammatory disease mechanisms, although further validation of these findings in larger cohorts is needed.

## 1 Introduction

Aquaporin‐4 (AQP4) antibody seropositive neuromyelitis optica spectrum disorder (AQP4+ NMOSD), myelin oligodendrocyte glycoprotein (MOG) antibody‐associated disease (MOGAD), and multiple sclerosis (MS) are immune‐mediated diseases of the central nervous system (CNS) with distinct pathophysiology. In AQP4+ NMOSD, binding of AQP4‐IgG to astrocytic AQP4 results in astrocyte injury by complement and cellular mechanisms, leading to an inflammatory response, blood–brain barrier disruption, and injury of oligodendrocytes and neurons [1]. MOG is expressed on the outer membrane of the myelin sheath, and MOGAD is thought to be an autoimmune oligodendrocytopathy [2]. MS represents a complex and pathologically heterogeneous entity wherein tissue damage results from a dynamic interplay between the immune system, glia, and neurons [3].

The distinct pathophysiology of AQP4+ NMOSD, MOGAD, and MS has raised the question of whether CSF cytokine signatures differ between these conditions and whether cytokines can be used as clinically relevant biomarkers. The IL‐6 receptor blocker satralizumab decreases relapse risk in AQP4+ NMOSD, while the IL‐6 receptor blocker tocilizumab has been effective for both NMOSD and MOGAD, supporting a possible role for IL‐6 in the pathogenesis of these disorders [4]. Several studies have reported elevated levels of cerebrospinal fluid (CSF) IL‐6 in AQP4+ NMOSD; however, few studies have examined cytokine profiles in MOGAD [5, 6, 7, 8, 9].

Both AQP4‐IgG and MOG‐IgG are predominantly of the IgG1 subclass and thus have the capacity to bind and activate the complement cascade through their Fc regions [10, 11]. Complement activation is a major mechanism of injury in AQP4+ NMOSD, and complement protein C5 inhibitors markedly reduce the frequency of relapses [4, 12]. However, it is unclear if complement is similarly relevant in MOGAD and whether CSF complement component levels are associated with disease activity or severity. Prior studies have reported elevated levels of serum complement components in MOGAD, as compared to MS, with many of these components appearing to have higher levels than in AQP4+ NMOSD [13, 14, 15]. However, the interpretation of serum complement measurements is challenging, since they may not accurately reflect intrathecal processes and may be influenced by systemic inflammation; this is particularly relevant for MOGAD, which frequently presents in association with an infectious prodrome [16]. Only a limited number of studies have quantified CSF complement components in MOGAD [15, 17].

A comprehensive evaluation of the complement pathway and cytokine profile during attacks in AQP4+ NMOSD, MOGAD and MS is lacking. In this study, our objective was to characterize and compare CSF complement components and cytokine profiles in CSF and plasma between AQP4+ NMOSD, MOGAD and MS, and to assess associations with neuro‐glial proteins and clinical features. We hypothesized that the antibody‐associated conditions would exhibit distinct cytokine profiles and that CSF complement components would be elevated in AQP4+ NMOSD and, perhaps to a lesser extent in MOGAD, suggesting activation of the complement pathway during acute attacks.

## 2 Methods

### 2.1 Study Population and Clinical Data

CSF and plasma samples were leveraged from our biorepository, which includes blood samples from patients with various neuroinflammatory disorders and healthy controls, as well as CSF specimens collected from patients undergoing lumbar punctures as part of their diagnostic evaluation at Johns Hopkins Hospital. Sample collection and processing is described in the Supporting Methods. We included samples from patients with MS, AQP4+ NMOSD, and MOGAD in two separate cohorts (CSF and plasma cohorts). For a subset of participants, paired CSF and plasma samples were available (collected within 2 days). MS participants fulfilled the 2024 revised McDonald diagnostic criteria and were classified into subtypes based on the Lublin classification [18, 19]. AQP4+ NMOSD participants had tested positive for AQP4‐IgG in the serum with commercial assays and fulfilled the 2015 International Panel for Neuromyelitis Optica Diagnosis criteria for AQP4‐IgG seropositive NMOSD [20]. MOGAD participants had tested positive for MOG‐IgG in the serum with commercial assays and fulfilled the 2023 International MOGAD panel diagnostic criteria [16].

For all neuroinflammatory disease groups, we included participants with inflammatory disease activity within 6 months prior to sample collection. For the AQP4+ NMOSD and MOGAD groups, this was defined as a symptomatic attack, and for the MS group, this was defined as a symptomatic attack or gadolinium‐enhancing lesions in the preceding 6 months. If multiple samples were available per participant, we selected the sample collected prior to initiation of immunotherapy and/or closest to an attack. Exclusion criteria for all disease groups included exposure to plasmapheresis in the preceding 3 months. Clinical data were collected retrospectively from chart review. If available, Expanded Disability Status Scale (EDSS) scores were recorded at nadir and at follow‐up > 6 months post‐attack (range 0.5–2.5 years), provided that no interval relapses occurred (in order to assess recovery from the index attack).

Control groups included individuals undergoing CSF sampling for assessment of non‐inflammatory conditions (non‐inflammatory controls; NIC) for the CSF cohort and healthy controls (HC) for the plasma cohort. NIC included patients with non‐inflammatory and non‐neurodegenerative neurologic conditions (migraine, idiopathic intracranial hypertension, functional neurologic disorder). HC were recruited among hospital staff and patient spouses.

### 2.2 Assays

Cytokines were measured in CSF and plasma using a multiplexed automated immunoassay system (ELLA; Bio‐Techne) per the manufacturer's instructions: IFN‐g, TNF‐a, IL‐6, IL‐10, IL‐12p70, IL‐17a, IL‐1b, IL‐1a, IL‐2, IL‐4, B‐cell survival factor (BAFF). Levels of the neuroglial proteins neurofilament light chain (NfL) and glial fibrillary acidic protein (GFAP) were also measured using the same multiplexed automated immunoassay system. CSF samples were analyzed undiluted, while plasma samples were analyzed at a 1:2 dilution. Each plate included samples representing all diagnostic groups. Analyte concentrations were measured in duplicates. Values were excluded if there was > 30% difference between duplicates within the reported linear range. Values below the lower limit of quantification (LLOQ) were replaced by LLOQ/√2 and values above the upper limit of quantification (ULOQ) were replaced by ULOQ. Analytes with > 75% values missing or below the LLOQ were not analyzed in downstream analyses. Quality control metrics for cytokine analytes are reported in Table S1.

The following complement components were measured in CSF: free C1q, C1s, C1 inhibitor (C1inh), C1s‐C1inh complex, C2, C2b, C3, C3a, C3d, C4, C4a, C5a, C5b‐9 (membrane attack complex) using a sandwich enzyme‐linked immunosorbent assay (ELISA), as described in detail in the Supporting Methods. Complement components were not measured in plasma.

### 2.3 Statistical Methods

Given that analyte concentrations exhibited positive skewness, values were logarithmically transformed for analyses. Comparisons between groups were performed using linear regression models; models were adjusted for age, as correlations with age were observed for several analytes in the control groups (Table S2). Comparisons between subgroups of interest (e.g., patients with optic neuritis [ON] vs. non‐ON attacks) were also performed with linear regression models. Correlations between cytokines and complement components with neuro‐glial proteins were evaluated using partial Pearson's correlation coefficients adjusted for age. Correlations between analytes in paired CSF and plasma were also evaluated with Pearson's correlation coefficients. Correlations between analytes and disability metrics (EDSS) were evaluated with Spearman's correlation coefficients, given disability metrics were not normally distributed. Statistical significance was defined as *p* < 0.05 (two‐tailed). For the primary analysis (comparison of each neuroinflammatory disease group to controls), *p*‐values were additionally corrected for multiple comparisons using the Benjamini‐Hochberg false discovery rate (FDR) procedure. For other analyses, given the exploratory nature of the study and a priori established research hypotheses [21], no correction for multiple comparisons was applied; consequently, the results should be interpreted cautiously as exploratory findings. Statistical analyses were performed with Stata (version 18) and graphs were made in R (version 4.5.0).

### 2.4 Ethical Considerations

The study was approved by the Institutional Review Board of Johns Hopkins University (NA_00003551, NA_00041259, IRB00242986) and written informed consent was obtained from all participants prior to study enrolment.

## 3 Results

### 3.1 Study Population

The CSF cohort included 19 participants with AQP4+ NMOSD, 29 with MOGAD, 36 with MS and 51 NIC. Demographics of the CSF cohort are summarized in Table 1. Mean (±SD) age at sampling was 42 ± 18 years for AQP4+ NMOSD, 35 ± 19 years for MOGAD, 36 ± 9 years for MS, and 37 ± 15 years for NIC. There were sex differences between groups consistent with the known demographic profile of each disease (higher proportion of females in the MS and AQP4+ NMOSD groups compared to MOGAD). Among the neuroinflammatory disease groups, the most common attack topography was ON for MOGAD (69%), myelitis for AQP4+ NMOSD (42%), and myelitis (31%) or multifocal presentations (31%) for MS. The attack represented the onset attack for 42% of AQP4+ NMOSD, 79% of MOGAD and 69% of MS participants. Median time from attack symptom onset to sampling was 12 days for AQP4+ NMOSD, 10 days for MOGAD and 27.5 days for MS. Most participants were not receiving maintenance disease‐modifying treatments at the time of sampling, except for 1 MS and 3 AQP4+ NMOSD participants. Among the NIC group, the majority (80%) had idiopathic intracranial hypertension or headache.

**TABLE 1:** Demographics of the cerebrospinal fluid cohort.

Group	AQP4+ NMOSD^a^	MOGAD^a^	MS	Non‐inflammatory controls^a^, ^b^
*N*	19	29	36	51
Age, years, mean ± SD	42.0 (±17.7)	34.5 (±19.4)	36.4 (±9.3)	36.5 (±14.5)
Female sex, *n* (%)	17 (89%)	14 (48%)	29 (81%)	37 (73%)
Race, *n* (%)				
White	4 (21%)	11 (38%)	19 (53%)	33 (65%)
Black	13 (68%)	9 (31%)	14 (39%)	14 (27%)
Other	2 (11%)	9 (31%)	3 (8%)	4 (8%)
Initial attack, *n* (%)	8 (42%)	23 (79%)	25 (69%)	—
Days since last attack, median (range)	12 (3–155)	10 (1–173)	27.5 (3–149)	—
Attack within 60 days, *n* (%)	13 (68%)	27 (93%)	25 (69%)	—
Preceding infection within 30 days, *n* (%)	1 (5%)	10 (34%)	2 (6%)	—
Attack type, *n* (%)	Myelitis 8 (42%) Optic neuritis 4 (21%) Brainstem 3 (16%) Multifocal 4 (21%)	Myelitis 3 (10%) Optic neuritis 20 (69%) Brainstem 1 (3%) Supratentorial 3 (10%) Multifocal 2 (7%)	Myelitis 11 (31%) Optic neuritis 4 (11%) Brainstem 5 (14%) Supratentorial 4 (11%) Multifocal 11 (31%) Radiologic 1 (3%)	—
Maintenance disease‐modifying treatment at time of lumbar puncture, *n*	None 16 Rituximab 2 Glatiramer acetate 1	None 29	None 35 Natalizumab 1	—
Exposure to steroids within 60 days, *n* (%)	10 (53%)	15 (52%)	16 (44%)	—
Exposure to IVIG within 60 days	0%	0%	0%	—
CSF cell count, median (range)^c^	6 (1–207)	4 (1–227)	4 (1–46)	1 (0–8)
CSF protein, median (IQR)	34 (24–47)	34 (25–55)	32 (27.5–40.5)	30 (22–43)
CSF‐specific oligoclonal bands^c^	4/16	4/29	34/34	—
Albumin quotient^c^, median (IQR), 10^−3^	4.9 (3.9–6.4)	4.7 (3.7–7.3)	4.2 (2.9–5.8)	4.1 (3.3–5.1)

Abbreviations: AQP4+ NMOSD, aquaporin‐4‐IgG seropositive neuromyelitis optica spectrum disorder; CSF, cerebrospinal fluid; MOGAD, myelin oligodendrocyte glycoprotein antibody associated disease; MS, multiple sclerosis; NIC, non‐inflammatory controls.

^a^ CSF complement components not available for 1 AQP4+ NMOSD, 6 MOGAD and 1 NIC.

^b^ Diagnoses: 8 functional neurological disorder, 2 healthy volunteers, 20 idiopathic intracranial hypertension, 21 headache/migraines.

^c^ CSF cell count not available for 2 MOGAD and 1 MS. CSF oligoclonal bands not available for 3 AQP4 and 2 MS. Albumin quotient not available for 2 AQP4, 4 MOGAD, 5 MS, 36 NIC.

The plasma cohort included 18 participants with AQP4+ NMOSD, 35 with MOGAD, 58 with MS, and 29 HC. Demographics of the plasma cohort are summarized in Table 2. Mean (±SD) age at sampling was 51 ± 19 years for AQP4+ NMOSD, 33 ± 14 years for MOGAD, 36 ± 9 years for MS, and 40 ± 12 years for HC. Similar to the CSF cohort, the most common attack topography was ON for MOGAD (66%), myelitis for AQP4+ NMOSD (50%), and multifocal presentations (31%) for MS. Median time from attack symptom onset to sampling was longer compared to the CSF cohort (46.5 days for AQP4+ NMOSD, 35 days for MOGAD and 45.5 days for MS). Most participants were not receiving maintenance immune treatments at the time of sampling, except for 2 MS, 6 MOGAD and 8 AQP4+ NMOSD participants.

**TABLE 2:** Demographics of the plasma cohort.

Group	AQP4+ NMOSD	MOGAD	MS	Healthy controls
*N*	18	35	58	29
Age, years, mean ± SD	50.6 (±19.1)	33.2 (±14.4)	36.3 (±8.5)	40.2 (±11.7)
Female sex, *n* (%)	15 (83%)	16 (46%)	45 (78%)	21 (72%)
Race, *n* (%)				
White	7 (39%)	16 (46%)	39 (67%)	18 (62%)
Black	8 (44%)	10 (29%)	16 (28%)	9 (31%)
Other	3 (17%)	9 (25%)	3 (5%)	2 (7%)
First attack, *n* (%)	6 (33%)	17 (49%)	30 (52%)	—
Days since last attack, median (range)	46.5 (3–170)	35 (2–173)	45.5 (3–149)	—
Attack within 60 days, *n* (%)	12 (67%)	22 (63%)	37 (64%)	—
Preceding infection within 30 days, *n* (%)	1 (6%)	4 (11%)	3 (5%)	—
Attack type, *n* (%)	Myelitis 9 (50%) Optic neuritis 5 (28%) Multifocal 2 (11%) Brainstem 2 (11%)	Myelitis 5 (14%) Optic neuritis 23 (66%) Brainstem 2 (6%) Cerebral 2 (6%) Multifocal 3 (9%)	Myelitis 12 (21%) Optic neuritis 9 (16%) Brainstem 8 (14%) Supratentorial 7 (12%) Multifocal 18 (31%) Radiologic 4 (7%)	—
Maintenance disease‐modifying treatment at time of blood draw, *n*	None 10 Rituximab 4 MMF 3 Glatiramer 1	None 29 Rituximab 2 MMF 1 IVIG 1 Combination 2	None 56 Natalizumab 1 Glatiramer 1	—
Exposure to steroids within 60 days, *n* (%)	11 (61%)	23 (66%)	35 (60%)	—
Exposure to IVIG within 60 days, *n* (%)	2 (11%)	2 (6%)	0 (0%)	—

Abbreviations: AQP4+ NMOSD, aquaporin‐4‐IgG seropositive neuromyelitis optica spectrum disorder; IVIG, intravenous immunoglobulin; MOGAD, myelin oligodendrocyte glycoprotein antibody‐associated disease, MS, multiple sclerosis, MMF, mycophenolate mofetil.

CSF and plasma samples collected within 2 days were available for 29 participants: 2 with AQP4+ NMOSD, 8 with MOGAD, and 19 with MS.

Analyte concentrations and ranges in CSF and plasma are presented in Tables S3 and S4. Complement components were only measured in CSF.

### 3.2 Comparisons Between Groups: Cytokines, NfL and GFAP

Overall findings are summarized in Figure 1. Comparisons of cytokine, NfL, and GFAP concentrations between groups are presented in Figure 2 and Table S5 for CSF, and Figure 3 and Table S6 for plasma.

**FIGURE 1:**
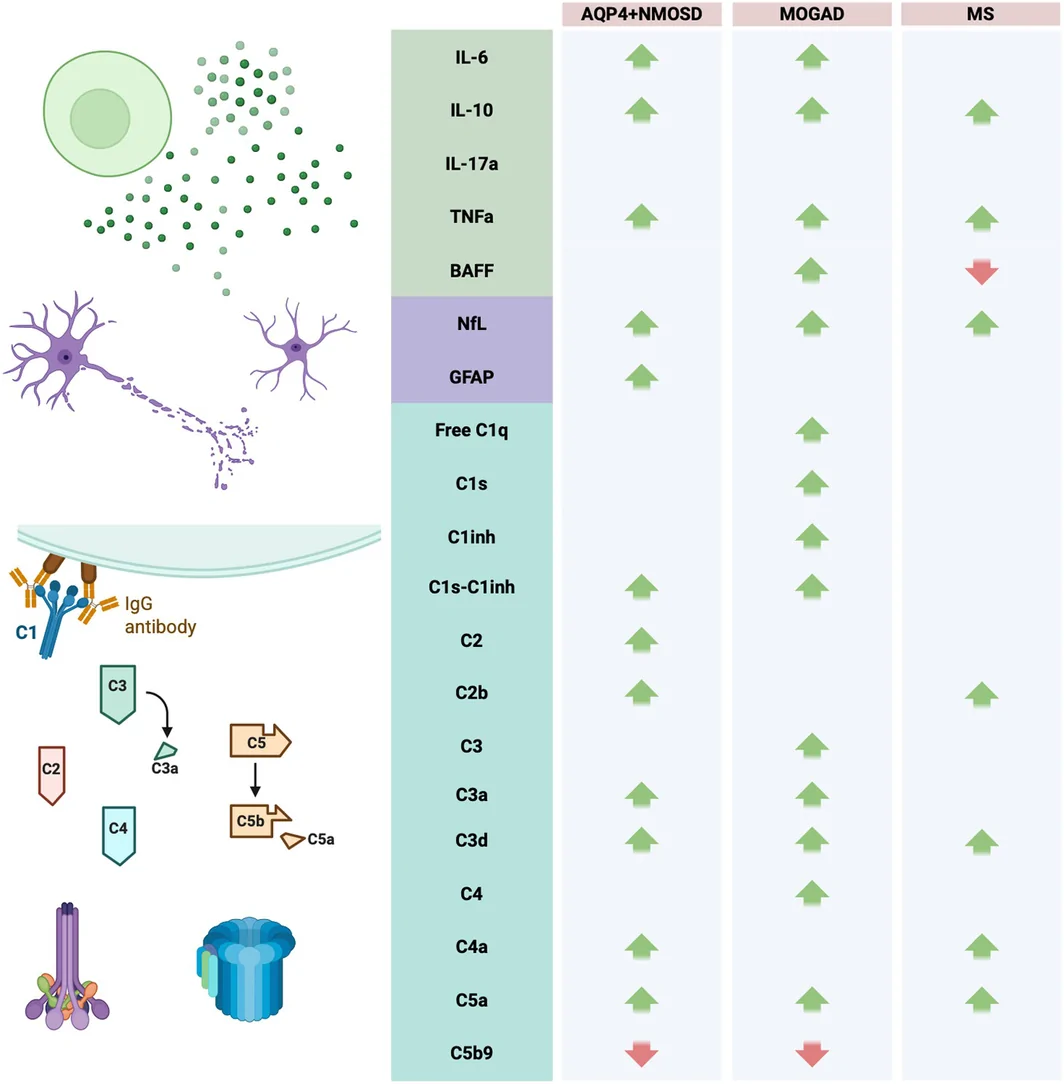
Overview of cytokine, complement, NfL and GFAP levels in AQP4+ NMOSD, MOGAD and MS relative to non‐inflammatory controls. Comparisons between groups were performed with linear regression models adjusted for age; statistical significance was defined as *p* < 0.05 (two‐tailed). (Figure created in BioRender).

**FIGURE 2:**
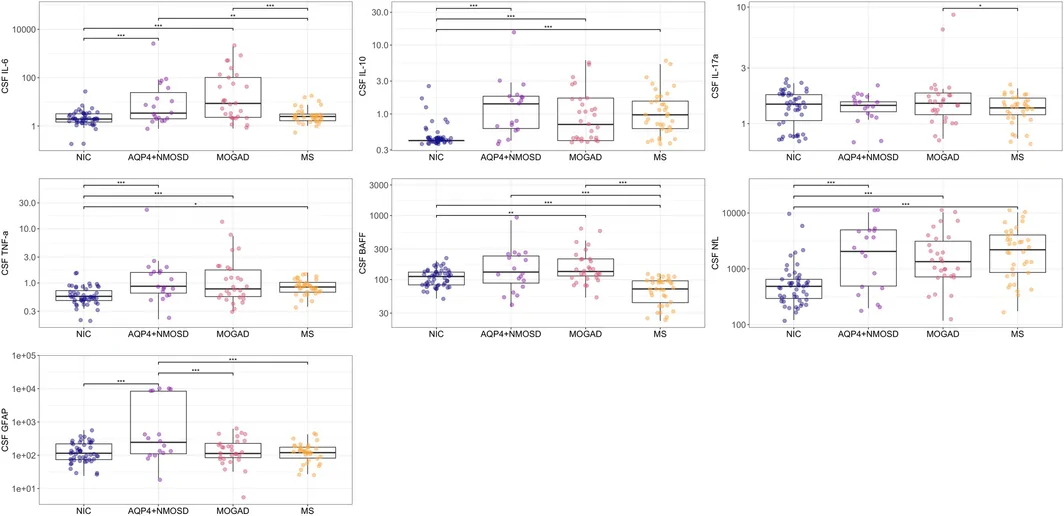
CSF cytokines, NfL and GFAP in AQP4+ NMOSD, MOGAD, MS and non‐inflammatory controls.

**FIGURE 3:**
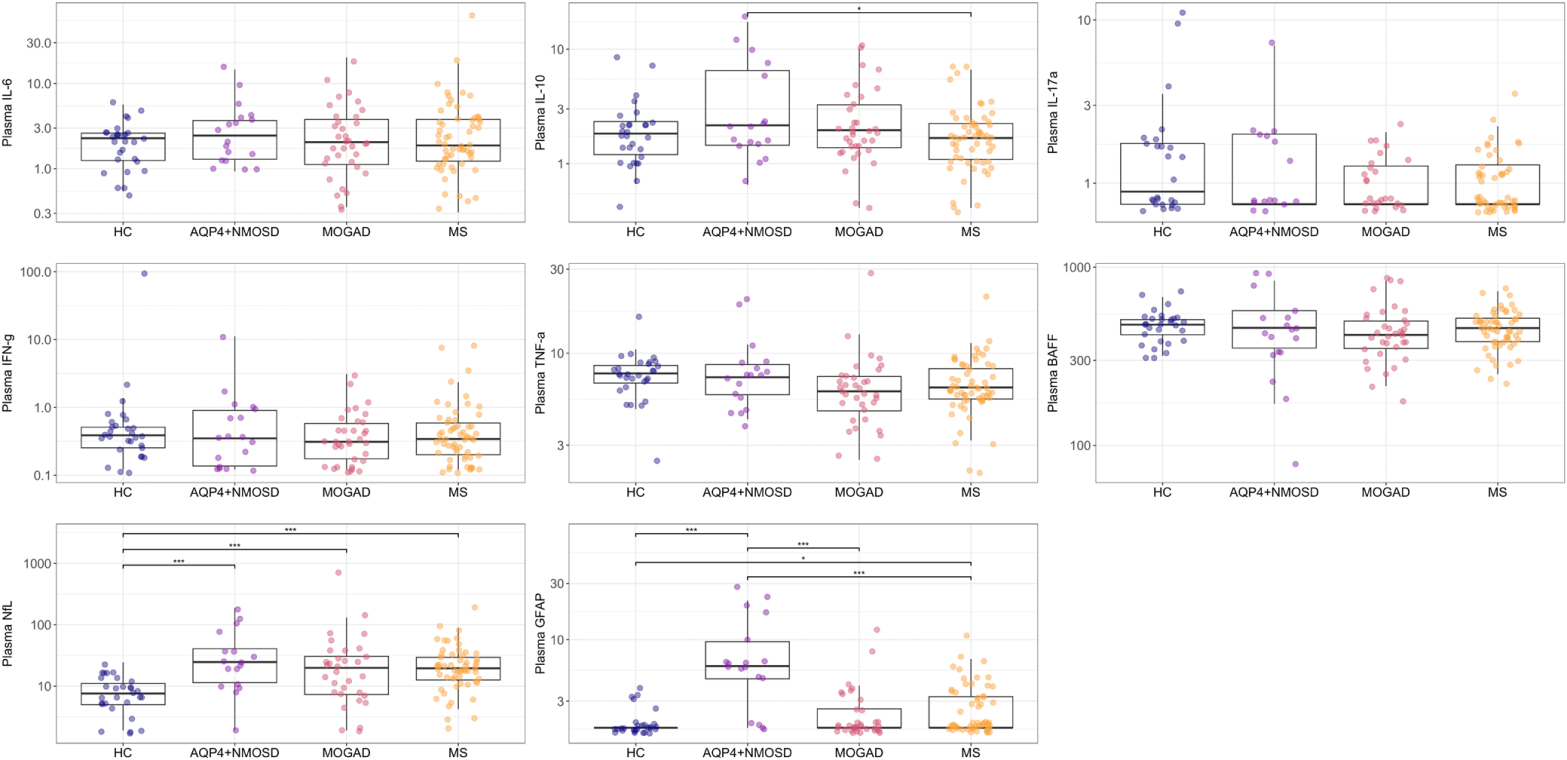
Plasma cytokines, NfL, and GFAP in AQP4+ NMOSD, MOGAD, MS, and healthy controls.

CSF IL‐10 and TNF‐a were higher in AQP4+ NMOSD, MOGAD, and MS compared to NIC (*p* ≤ 0.01 for all) but did not differ between neuroinflammatory disease groups. CSF IL‐6 was elevated in AQP4+ NMOSD and MOGAD (*p* < 0.001) compared to NIC and MS but did not differ between MS and NIC, nor between AQP4+ NMOSD and MOGAD. There were no significant differences in CSF IL‐17a between groups. CSF BAFF was higher in MOGAD compared to NIC (*p* = 0.001); a similar trend was observed in AQP4+ NMOSD but did not achieve statistical significance (*p* = 0.07), and there was no difference between AQP4+ NMOSD and MOGAD. CSF BAFF was lower in MS compared to NIC, AQP4+ NMOSD and MOGAD (*p* < 0.001 for all). CSF NfL was higher in AQP4+ NMOSD, MOGAD and MS compared to NIC (*p* < 0.001 for all). CSF GFAP was higher in AQP4+ NMOSD compared to all other groups (*p* < 0.001 for all). Sensitivity analyses yielded similar findings when excluding participants receiving maintenance disease‐modifying treatments at the time of sampling and when restricting to samples collected within 60 days of attack symptom onset (data not shown).

There were no meaningful differences in plasma cytokine levels between HC and disease groups. Plasma NfL was higher in AQP4+ NMOSD, MOGAD and MS (*p* < 0.001 for all) compared to HC but did not differ between disease groups. Plasma GFAP was higher in AQP4+ NMOSD compared to all other groups (*p* < 0.001 for all). Plasma GFAP was higher in MS (*p* = 0.03) compared to HC. Findings were largely similar in sensitivity analyses restricting to samples collected within 60 days of attack symptom onset (data not shown).

### 3.3 Correlation Between CSF and Plasma Analytes

Paired CSF and plasma samples collected within 2 days were available for 29 participants: 2 AQP4+ NMOSD, 8 MOGAD, 19 MS. No correlations were found between CSF and plasma levels of IL‐6, IL‐10, IL‐17a, TNF‐a (Table S7). Moderate correlations were found between CSF and plasma BAFF (*r* = 0.51, *p* = 0.005) and GFAP (*r* = 0.60, *p* < 0.001), while a strong correlation was found between CSF and plasma NfL (*r* = 0.79, *p* < 0.001).

### 3.4 Comparisons Between Groups: Complement Components

Overall findings are summarized in Figure 1. Comparisons of CSF complement component levels between groups are presented in Figure 4 and Table S5.

**FIGURE 4:**
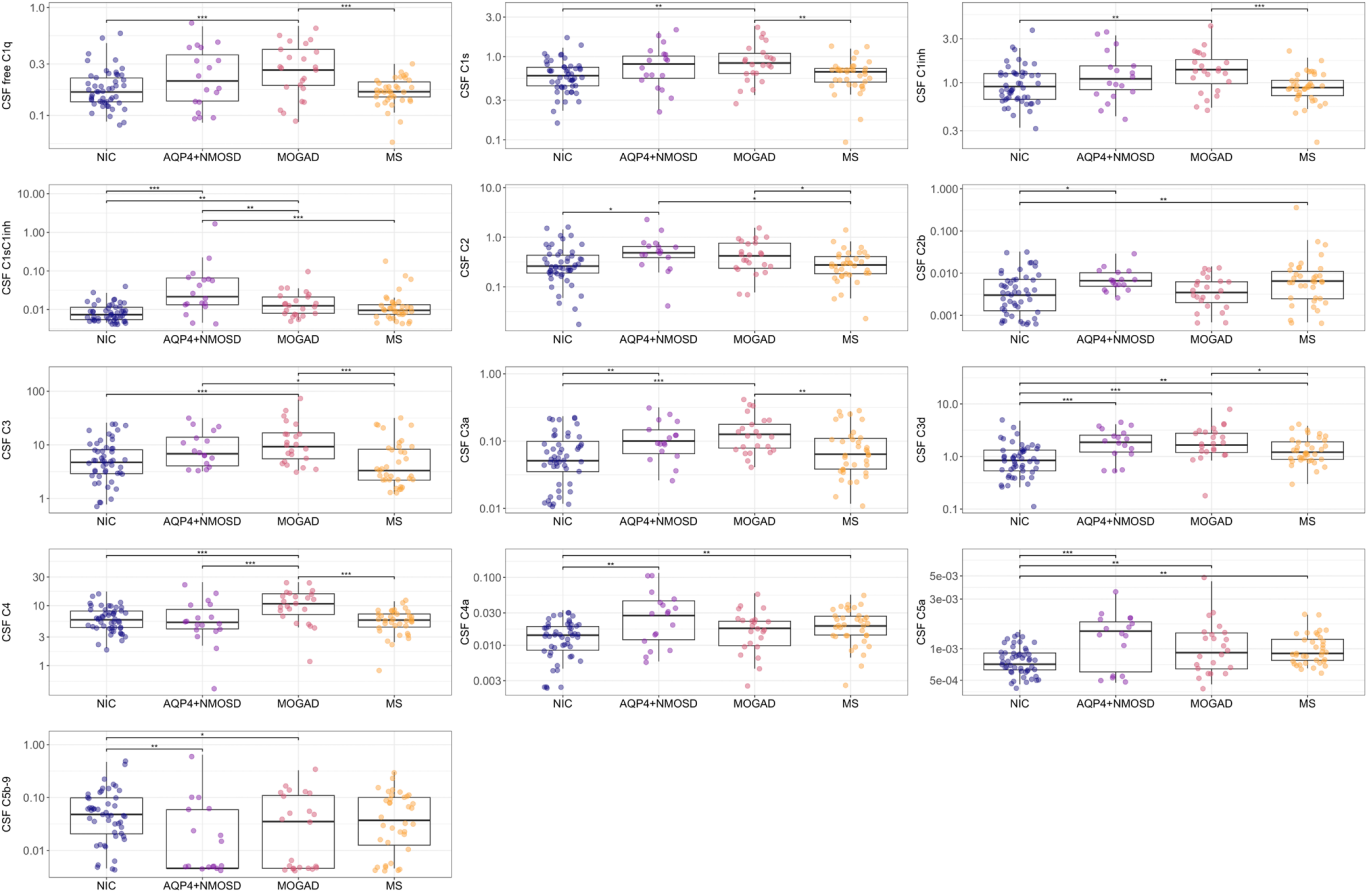
CSF complement components in AQP4+ NMOSD, MOGAD, MS and non‐inflammatory controls.

Several CSF complement components, including C1s‐C1inh, C2, C2b, C3a, C3d, C4a, C5a, were higher in AQP4+ NMOSD compared to NIC (*p* ≤ 0.02 for all). CSF free C1q, C1s, C1inh, C1s‐C1inh, C3, C3a, C3d, C4, C5a, were elevated in MOGAD compared to NIC (*p* ≤ 0.01 for all). There were no significant differences between AQP4+ NMOSD and MOGAD, except for C4, which was higher in MOGAD (*p* < 0.001), and C1s‐C1inh, which was higher in AQP4+ NMOSD (*p* = 0.009). In MS, specific activation products including CSF C2b, C3d, C4a, and C5a were higher compared to NIC (*p* ≤ 0.01 for all). Several complement components were higher in AQP4+ NMOSD (C1s‐C1inh, C2, C3) and in MOGAD (free C1q, C1s, C1inh, C2, C3, C3a, C3d, C4), as compared to MS. Sensitivity analyses yielded similar findings when excluding participants receiving maintenance disease‐modifying treatments at the time of sampling and when restricting to samples collected within 60 days of attack symptom onset (data not shown). CSF C5b‐9 was lower in AQP4+ NMOSD and MOGAD compared to NIC but lost statistical significance after FDR correction for MOGAD and in sensitivity analyses restricted to samples collected within 60 days of attack symptom onset for both AQP4+ NMOSD and MOGAD.

### 3.5 Correlation of CSF Cytokines and Complement Components With NfL and GFAP

Correlations of CSF cytokines and complement components with CSF neuroglial proteins in AQP4+ NMOSD, MOGAD and MS are presented in Table S8 and Figures 5 and 6. In AQP4+ NMOSD, CSF GFAP was positively correlated with CSF cytokines including IL‐6, IL‐10, TNF‐a, and BAFF. CSF NfL was positively correlated with CSF TNF‐a. CSF GFAP was positively correlated with complement components including free C1q, C1s‐C1inh and C4a. In MOGAD, both CSF NfL and GFAP were positively correlated with several CSF complement components including free C1q, C1inh, C1s‐C1inh, C2, C2b, C3d, C4, C4a. Additionally, CSF GFAP was positively correlated with TNF‐a and C1s. In MS, CSF NfL was positively correlated with BAFF and C4a, while CSF GFAP was correlated with TNF‐a and C2.

**FIGURE 5:**
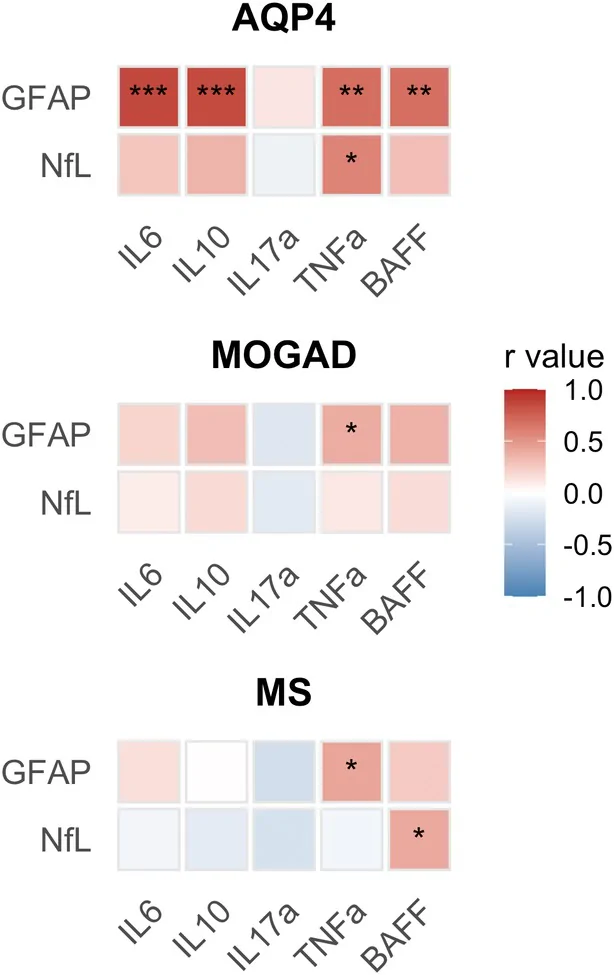
Correlation of CSF cytokines with NfL and GFAP in AQP4+ NMOSD, MOGAD and MS.

**FIGURE 6:**
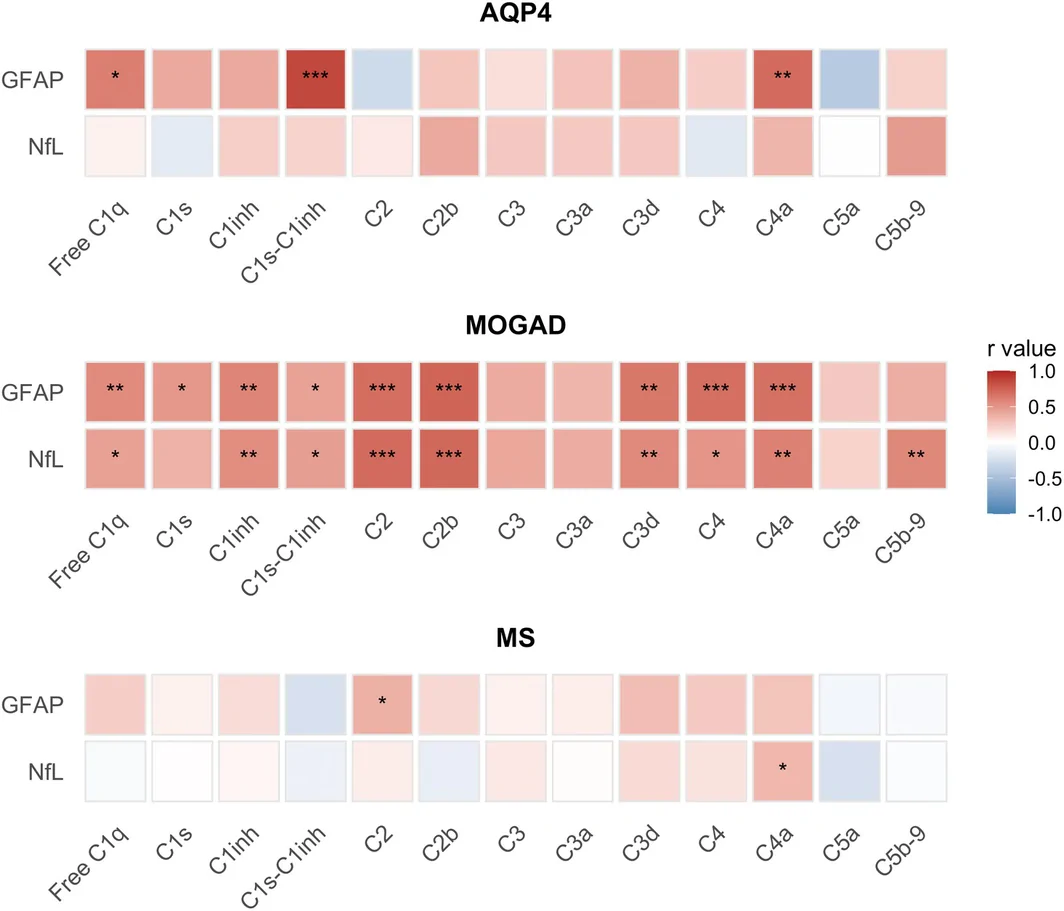
Correlation of CSF complement components with NfL and GFAP in AQP4+ NMOSD, MOGAD, and MS.

### 3.6 Associations of CSF Analytes With Clinical Characteristics

Associations of CSF analytes with clinical characteristics in AQP4+ NMOSD are presented in Table S9. In AQP4 + NMOSD, NfL and GFAP were higher in participants with non‐ON (*n* = 15) compared to ON (*n* = 4) attacks (2.10 ± 0.71, *p* = 0.01; 2.55 ± 1.20, *p* = 0.05 respectively). No significant differences were observed for other analytes between attack locations. No differences were observed between participants with samples collected within 60 days of attack symptom onset (*n* = 13) and those collected between 60 and 180 days (*n* = 6). Correlations were found between EDSS at nadir and CSF BAFF (rho = 0.56, *p* = 0.033), GFAP (rho = 0.52, *p* = 0.05), free C1q (rho = 0.58, *p* = 0.032), C1s (rho = 0.54, *p* = 0.047), C1s‐C1inh (rho = 0.64, *p* = 0.017) and C4a (rho = 0.59, *p* = 0.027). There were insufficient participants to evaluate correlations with follow‐up EDSS. Participants with steroid exposure within 60 days of sampling had lower CSF IL‐10 and C4a compared to those without; otherwise, no major differences were observed by steroid exposure status.

Associations of CSF analytes with clinical characteristics in MOGAD are presented in Table S10. In MOGAD, IL‐6, IL‐10, TNF‐a, BAFF, free C1q, C1inh, C1s‐C1inh, C2b and C4a were higher in participants with non‐ON (*n* = 9) compared to ON (*n* = 20) attacks. Comparisons between participants with samples collected within 60 days of attack symptom onset and those collected between 60 and 180 days could not be performed since all participants except two had samples collected within 60 days. Correlations were found between EDSS at nadir and CSF IL‐10 (rho = 0.45, *p* = 0.024), TNF‐a (rho = 0.45, *p* = 0.027), free C1q (rho = 0.45, *p* = 0.049), C1inh (rho = 0.47, *p* = 0.039) and C4a (rho = 0.48, *p* = 0.033), as well as between C2 and follow‐up EDSS (rho = 0.55, *p* = 0.045). Participants with steroid exposure within 60 days of sampling had higher CSF C3a compared to those without; otherwise, no major differences were observed by steroid exposure status.

Associations of CSF analytes with clinical characteristics in people with MS are presented in Table S11. MS samples collected within 60 days of attack symptom onset had higher CSF IL‐10 and lower CSF BAFF compared to those with samples collected between 60 and 180 days post‐attack (0.53 ± 0.25, *p* = 0.044; −0.36 ± 0.18, *p* = 0.05 respectively). No correlations were found between CSF analytes and EDSS at nadir or follow‐up EDSS. No major differences in analytes were observed by steroid exposure status.

## 4 Discussion

In this study, we investigated complement components and cytokine profiles in AQP4+ NMOSD, MOGAD and MS, with the aim to gain insight into their potential role in disease immunopathogenesis. Our findings highlight several key observations. With regards to CSF cytokines, IL‐10 and TNF‐a were elevated across all neuroinflammatory disease groups, while IL‐6 was specifically elevated in AQP4+ NMOSD and MOGAD. In contrast, there were no meaningful differences in plasma cytokines across disease groups. With regards to CSF complement findings, there was a widespread increase of complement components in AQP4+ NMOSD and MOGAD, suggesting complement pathway activation and supporting a potential role for complement‐mediated injury in both conditions.

Our findings strengthen the view of IL‐6 as a central player in the pathophysiology of AQP4+ NMOSD and MOGAD. IL‐6 plays a critical role in the differentiation of B cells to plasma cells and of naïve T‐cells to inflammatory Th17 cells, which aid in B‐cell activation. Prior studies have suggested that AQP4+ NMOSD is a Th2‐ and Th17‐dominant disease, and IL‐6 has been consistently found to be elevated compared to MS and controls across several studies [5, 6, 7, 22]. In AQP4+ NMOSD, IL‐6 is thought to play multiple roles in pathogenesis by supporting plasmablast survival, promoting anti‐AQP4 antibody production and enhancing proinflammatory T‐lymphocyte activity. Anti‐IL‐6 treatment with satralizumab is an approved therapeutic strategy, supporting the relevance of this pathway in AQP4+ NMOSD [4]. Elevated CSF IL‐6 levels in both MOGAD and AQP4+ NMOSD in this study may indicate overlapping pathogenic mechanisms involving IL‐6 and are concordant with prior studies reporting elevated CSF IL‐6 during the acute phase in MOGAD [8, 23, 24]. Higher CSF IL‐6 concentrations in MOGAD were associated with poorer neurological recovery in a study by Uzawa et al. [8]. Kaneko et al. reported higher levels of IL‐6, IL‐8, IFN‐g and IL‐10 in MOGAD compared to MS, while there were no differences from AQP4+ NMOSD [9]. Taken together, these findings suggest that the CSF cytokine profile may be distinct in autoantibody‐mediated demyelination compared to MS, with IL‐6 playing an important role. Observational studies suggest that anti‐IL‐6 therapy is effective in MOGAD and a Phase III clinical trial of satralizumab, approved for AQP4+ NMOSD, is ongoing in relapsing MOGAD [25, 26]. Although prior studies have identified differences in blood cytokines between MOGAD and MS, the present work did not detect differences in plasma cytokines between neuroinflammatory diseases and controls [27, 28, 29]. Additionally, plasma cytokines exhibited poor correlations with CSF levels; however, the number of available paired samples was relatively small.

An important finding in our study was the observation of elevated B‐cell‐associated cytokine BAFF in MOGAD. A similar effect size was observed in AQP4+ NMOSD, but the difference did not reach statistical significance. A correlation between BAFF levels and EDSS at nadir was noted for AQP4+ NMOSD, and BAFF was higher in MOGAD non‐ON presentations. Our findings are in agreement with prior reports showing elevations in B‐cell related cytokines in pediatric MOG‐IgG positive compared to MOG‐IgG negative demyelinating syndromes [24]. BAFF is produced primarily by innate immune cells, binds to receptors on B cells and regulates the survival, homeostasis, proliferation, and activation of B cells; elevated BAFF levels likely indicate enhanced B cell activation and recruitment [30]. On the contrary, CSF BAFF was lower in MS compared to NIC. Additionally, CSF BAFF was lower in people with MS with samples collected earlier after attack symptom onset. Consistent with our findings, several studies have reported low CSF BAFF in MS at disease onset [31, 32, 33, 34]. Our data suggest that CSF BAFF levels are reduced in the early acute phase of an MS relapse, which may reflect local utilization of this factor by intrathecally recruited B cells. These observations may point to differences in B‐cell immunobiology between antibody‐mediated demyelinating diseases (AQP4+ NMOSD, MOGAD) and MS, although their exact implications remain uncertain. The role of B‐cell cytokines during the course of MS is complex [30]. In a Th17 adoptive transfer experimental autoimmune encephalomyelitis (EAE) model that recapitulates aspects of cortical and leptomeningeal inflammation in MS, overexpression of BAFF reduced EAE severity, while co‐treatment of EAE mice with anti‐BAFF and anti‐CD20 prevented the benefits of anti‐CD20 on demyelination and axonal loss [35]. In human studies, atacicept, a fusion protein which blocks the BAFF/APRIL system and suppresses B‐cell function, was unexpectedly associated with increased clinical disease activity in a Phase 2 clinical trial in relapsing MS [36]. It has been proposed that BAFF may exert neuroprotective effects in MS; however, current data with regards to its exact role remain inconclusive.

Another relevant observation of our study was the suggestion of widespread activation of complement pathways in AQP4+ NMOSD and MOGAD, as demonstrated by elevated levels of complement components across the complement cascade in both conditions. Several of the elevated components are linked to the classical pathway, while others overlap with the lectin pathway (e.g., C2, C4), and some represent terminal products common to all pathways (e.g., C3a, C5a). Elevated early classical pathway components in AQP4+ NMOSD (C1s‐C1inh) and MOGAD (free C1q, C1s, C1inh) support antibody‐mediated complement activation; however, contributions from other pathways cannot be excluded. Complement activation is a known mechanism of injury in AQP4+ NMOSD, and complement C5 inhibitors eculizumab and ravulizumab are highly effective in suppressing relapses [4, 12]. Prior studies have reported elevated CSF complement components in AQP4+ NMOSD; Kuroda et al. reported elevated CSF C5a in AQP4+ NMOSD during attacks, while Zelek et al. found increased CSF complement components, though most patients were not studied during acute attacks [37, 38]. In contrast, the role of complement in MOGAD is not well defined. While our findings raise the possibility of a pathogenic contribution, these data alone are insufficient to support this conclusion and may instead reflect secondary effects of acute inflammation rather than a primary disease mechanism. While we did not observe major differences in complement components between AQP4+ NMOSD and MOGAD, distinct complement components were associated with levels of CSF neuroglial proteins in each disorder, which may indicate disease‐specific mechanisms linking complement activation to neuro‐axonal and/or astrocytic injury. We additionally found modest correlations between some complement components and EDSS at nadir for AQP4+ NMOSD and MOGAD. In a prior study, Villacieros et al. evaluated CSF levels of complement components in AQP4+ NMOSD, MOGAD, and MS, and similarly observed elevations in several components in MOGAD compared to MS, but no major differences relative to AQP4+ NMOSD; however, no control group was included [15]. In the same study, complement activation products and regulators were associated with EDSS at follow‐up in MOGAD. Furlan et al. found elevated C5a levels, and Kaneko et al. found increased CSF C3a and C5a levels in MOGAD and AQP4+ NMOSD compared to MS [17, 39]. A noteworthy observation in our study is that CSF C5b‐9 levels were lower in both AQP4+ NMOSD and MOGAD compared to NIC. This may reflect increased complement consumption due to activation and membrane attack complex deposition within CNS lesions rather than diffusely in the CSF compartment or may be attributable to the lability and rapid degradation of C5b‐9. In contrast, Kaneko et al. reported higher C5b‐9 in AQP4+ NMOSD compared to MOGAD, with no differences compared to MS and NIC; however, interpretation of those results is limited by the small control group sample sizes [17].

Similar to AQP4‐IgG, MOG‐IgG is predominantly of the IgG1 subclass and may activate the complement cascade. MOG‐IgG has been demonstrated to induce complement‐dependent cytotoxicity (CDC), antibody‐dependent cellular cytotoxicity (ADCC), and antibody‐dependent cellular phagocytosis (ADCP), but likely less efficient CDC than AQP4‐IgG [40, 41, 42]. In contrast to the hypothesis of a pathogenic contribution of complement in MOGAD, Macrini et al. postulated that CDC is not a major mechanism in MOGAD, since MOG‐IgG appears to require bivalent binding to MOG monomers and C1q, an initiator of the classical complement pathway, poorly binds to MOG‐IgG‐MOG complexes [43]. In murine models of MOGAD involving EAE with passive transfer of pathogenic MOG‐IgG, demyelination was mediated by both complement and Fc‐receptor‐mediated effects about equally [44]. In pathologic studies, deposition of complement has been reported in active white matter lesions of MOGAD patients, but less consistently compared to AQP4+ NMOSD [45, 46]. In a recent histopathological study, distinct patterns of complement deposition were identified in NMOSD, MOGAD and MS lesions [47]. In MOGAD, C4d deposition was detected in the majority of perivenous demyelinating lesions and colocalized with myelin, suggesting that perivascular myelin sheath disruption involves complement in the acute lesions. However, C9neo deposition varied: some lesions showed limited C9neo deposition with relatively preservation of oligodendrocytes and others showed marked deposition of C9neo and marked depletion of oligodendrocytes, suggesting inter‐individual differences in membrane attack complex formation in MOGAD. Ultimately, the contribution of complement to MOGAD pathogenesis remains uncertain, and further research is required to define its pathogenic relevance and therapeutic potential.

In MS, the role of complement is complex. Neuropathologic studies have shown deposition of complement and immunoglobulins in MS lesions, and complement activation has been implicated in synaptic pathology and chronic compartmentalized inflammation [48, 49, 50, 51]. In the present study, we noted elevated levels of CSF complement activation products C2b, C3d, C4a, and C5a in MS compared to NIC, while C4a exhibited a positive correlation with NfL. In a prior study, Oechtering et al. reported elevated CSF C4a and C3a but not C5a across all MS stages, and both C4a and C3a exhibited correlations with EDSS and NfL [52]. In a follow‐up study by the same group, higher C4a, C3a, C3, C5a, and C5 were linked to accelerated brain atrophy, higher C4a and C3a were linked to longitudinally greater T2 lesion volumes, and higher C3a and C5a were linked to increased odds of contrast‐enhancing and paramagnetic rim lesions [53]. It has therefore been hypothesized that aberrant CSF complement activation may be associated with structural brain damage relevant to disability accumulation in MS. Future studies are needed to further elucidate the mechanistic pathways responsible for the associations reported in the current and prior studies.

In the present work, we also examined the neuro‐glial proteins NfL and GFAP, both of which have emerged as biomarkers for MS, AQP4+ NMOSD and MOGAD. CSF and plasma NfL were elevated in AQP4+ NMOSD, MOGAD and MS during the active phases but did not differentiate between neuroinflammatory disease groups. GFAP was significantly higher in AQP4+ NMOSD in both CSF and plasma compared to MS and MOGAD, consistent with known disease pathophysiology involving astrocytic injury. Attack‐related increase in GFAP in AQP4+ NMOSD is well documented and has been shown to correlate with attack severity [54]. Plasma GFAP was also found to be elevated in MS; GFAP has been proposed as a biomarker of progressive biology in MS [55]. While GFAP was not elevated in MOGAD, we did observe associations between GFAP and complement components. Prior studies with regards to the role of GFAP as a biomarker in MOGAD are conflicting; some studies have reported elevated serum GFAP in MOGAD compared to MS and controls (particularly during attacks), while others have not observed meaningful elevations [56, 57, 58, 59]. Contrary to AQP4+ NMOSD, astrocytes are largely spared in MOGAD and dystrophic astrocytes indicative of astrocytopathy are not observed [46, 60].

This study has several notable limitations. Our sample size was relatively small, particularly for AQP4+ NMOSD, although this is an expected limitation given the rarity of conditions studied and the challenges associated with collection of samples during acute presentations. Participants were recruited from a single referral center and may therefore differ from patients evaluated in other settings. Validation of our findings in larger, independent cohorts would be of significant interest. Additionally, although we intended to examine samples during acute attacks, we included samples collected up to 6 months from attack symptom onset. This window may have been too broad, since cytokines and complement components are likely highly dynamic during and after acute attacks. Nevertheless, a sensitivity analysis restricted to samples collected within 60 days of attack symptom onset yielded largely similar results. A proportion of patients had received corticosteroids before sampling, although the vast majority were not on maintenance immunotherapy. While we did not observe major differences between patients with and without corticosteroid exposure before sampling, treatment effects cannot be fully excluded. Analyses restricted to treatment‐naïve, very acute samples (i.e., collected within days of attack symptom onset) were not feasible due to sample size constraints. Furthermore, the plasma analyses warrant cautious interpretation. Although we did not observe meaningful differences in plasma analytes between controls and disease groups, it is important to note that plasma samples were more often collected later after attack onset compared to CSF (> 30 days), and a greater proportion of patients were on immunotherapy at the time of plasma collection. In addition, the number of paired CSF and plasma samples was limited (approximately 1/3 of the cohort), which limits conclusions regarding comparisons between plasma and CSF analyte levels. Moreover, several potential confounders cannot be fully accounted for retrospectively, including differences between cohorts in time from symptom onset to sample collection, as well as differences in demographic and clinical characteristics, some of which are expected given known differences between the diseases. Our study was underpowered for multivariable analyses incorporating these factors, and residual confounding cannot be excluded. Additionally, we only examined a single time point per patient; longitudinal data are needed to determine analyte dynamics and within‐patient changes over time. Some cytokines were undetectable with many samples falling below the LLOQ, preventing further analyses of those analytes. Lastly, our NfL measurements were performed using the ELLA platform rather than other platforms commercially used in this space such as single molecule array, however these assays exhibit excellent correlation [61].

In conclusion, we report a comprehensive evaluation of the complement pathway and cytokine profiles in AQP4+ NMOSD, MOGAD and MS. Our findings suggest distinct CSF cytokine profiles in AQP4+ NMOSD, MOGAD and MS, and widespread activation of the classical complement pathway in AQP4+ NMOSD and MOGAD. We propose that characterizing patterns of complement activation and cytokine profiles may facilitate differential diagnosis, help identify potential novel therapeutic targets and provide further insights into the CNS microenvironment, thereby advancing our understanding of underlying pathophysiological mechanisms in neuroinflammatory disorders.

## Author Contributions

Angeliki G. Filippatou, Kathryn C. Fitzgerald, Peter A. Calabresi, Carlos A. Pardo, and Elias S. Sotirchos contributed to the conception and design of the study. Angeliki G. Filippatou, David Acero‐Garces, Georgios Gakis, Matthew D. Smith, Yasmin Resto, Maria Reyes‐Mantilla, Haiwen Chen, Isabella Hernandez, Yana Said, Andrea Salazar‐Camelo, Christina Huynh, Alessia Tassoni, Ted Yednock, Rick Artis, Yaisa Andrews‐Zwilling, Scott D. Newsome, Shiv Saidha, Pavan Bhargava, Ellen M. Mowry, Kathryn C. Fitzgerald, Peter A. Calabresi, Carlos A. Pardo, and Elias S. Sotirchos contributed to data acquisition, analysis, and/or interpretation. Angeliki G. Filippatou and Elias S. Sotirchos contributed to drafting the text and/or preparing the figures. All authors reviewed and edited the final manuscript. Angeliki G. Filippatou and Elias S. Sotirchos had access to raw data and final responsibility for the decision to submit for publication.

## Funding

This work was supported by the National Institutes of Health (R25NS065729 to Angeliki G. Filippatou, K23NS117883 to Elias S. Sotirchos, R01NS123712 to Carlos A. Pardo, K12NS098482‐08 to Haiwen Chen), the National Multiple Sclerosis Society (FP‐2307‐41984 to Angeliki G. Filippatou, RG‐1904‐33834 to Elias S. Sotirchos), the Sumaira Foundation, the Bart McLean Fund for Neuroimmunology Research, the Siegal Rare Neuroimmune Association and the Caring Friends for NMO Research Fund. Complement assays were performed by Annexon Biosciences.

## Disclosure

Angeliki G. Filippatou, Susana Dominguez‐Penuela, David Acero‐Garces, Georgios Gakis, Matthew D. Smith, Yasmin Resto, Maria Reyes‐Mantilla, Isabella Hernandez, Yana Said, Andrea Salazar‐Camelo and Carlos Pardo report no relevant disclosures. Haiwen Chen is site PI for contract research for Roche/Genentech. Christina Huynh, Alessia Tassoni and Ted Yednock are employees with and have equity ownership in Annexon Biosciences. Rick Artis and Yaisa Andrews‐Zwilling are employees with Annexon Biosciences. Scott D. Newsome has received consultant fees for scientific advisory boards from Genentech, Novartis, Sanofi and TG Therapeutics, is the study lead PI for a Roche clinical trial program,and has received research funding (paid directly to institution) from Biogen, Roche, Lundbeck, Genentech, Sanofi and Kyverna Therapeutics. Shiv Saidha has received consulting fees from Medical Logix for the development of CME programs in neurology and has served on scientific advisory boards for Biogen, Novartis, Sanofi, Genentech Corporation, Immunic therapeutics, Horizon, Amgen, Clene Pharmaceuticals & ReWind therapeutics. He has performed consulting for Novartis, Genentech Corporation, JuneBrain LLC, Innocare Pharma, Kiniksa pharmaceuticals, Setpoint Medical and Lapix therapeutics. He is the PI of investigator‐initiated studies funded by Genentech Corporation, TG therapeutics, Biogen, and Novartis. He has received equity compensation for consulting from JuneBrain LLC and Lapix therapeutics. He was also the site investigator of trials sponsored by MedDay Pharmaceuticals, Clene Pharmaceuticals, Novartis and Lapix therapeutics. Ellen M. Mowry serves as PI for investigator‐initiated studies from Biogen and Genentech, receives royalties for editorial duties on UpToDate and has received consulting fees from Octave and SetPoint Medical. Pavan Bhargava has received honoraria from Genentech and EMD Serono, and grants from Genentech, EMD Serono, GSK, and Amylyx Pharmaceuticals. Kathryn C. Fitzgerald has received consulting fees from Set Point Medical. Peter A. Calabresi has received consulting fees from Lilly, Biogen, and Novartis; and is PI on grants to Johns Hopkins from Genentech and SetPoint. Elias S. Sotirchos has consulted for Alexion, Amgen, TG Therapeutics, and Roche/Genentech, is site principal investigator for studies funded by Alexion, Roche/Genentech, UCB, and Ad Scientiam, and is principal investigator for an investigator‐initiated study funded by Astoria Biologica.

## Conflicts of Interest

Alessia Tassoni, Christina Huynh, and Ted Yednock are employees of and have equity ownership in Annexon Biosciences. Rick Artis and Yaisa Andrews‐Zwilling are employees of Annexon Biosciences.

## Supporting information


Supplementary Methods.
**Table S1:** Quality control metrics for cytokine analytes.
**Table S2:** Associations of analytes with demographics (age, sex) in non‐inflammatory controls in CSF and healthy controls in plasma.
**Table S3:** Analyte concentrations and ranges in CSF.
**Table S4:** Analyte concentrations and ranges in plasma.
**Table S5:** Comparison between groups for the CSF cohort.
**Table S6:** Comparison between groups for the plasma cohort.
**Table S7:** Correlations between CSF and plasma levels of cytokines and neuroglial proteins.
**Table S8:** Correlations of CSF cytokines and complement proteins with CSF neuroglial proteins in AQP4+ NMOSD, MOGAD and MS.
**Table S9:** Associations of CSF analytes with clinical characteristics in AQP4+ NMOSD.
**Table S10:** Associations of CSF analytes with clinical characteristics in MOGAD.
**Table S11:** Associations of CSF analytes with clinical characteristics in MS.

## Data Availability

The data that support the findings of this study are available on request from the corresponding author. The data are not publicly available due to privacy or ethical restrictions.
