# Supplementary material for: Investigation of Complement Activation and Cytokine Signatures in AQP4+ NMOSD, MOGAD, and MS
Source: Ann Clin Transl Neurol. 2026 Oct 21:10.1002/acn3.70549. Online ahead of print. doi: 10.1002/acn3.70549 (PMC13687446; doi:10.1002/acn3.70549)
Supplement: Supplementary file 1 [file ACN3-9999-0-s001.docx]

**SUPPLEMENTARY MATERIAL**

**SUPPLEMENTARY METHODS**

Sample collection and processing

Cerebrospinal fluid (CSF) samples were collected via lumbar puncture in sterile tubes without additives. Samples were centrifuged for 10 min at 1000 rpm to separate the cellular and fluid phases. The supernatant was then collected and aliquoted. Blood samples were collected by venipuncture in tubes anticoagulated with EDTA and were centrifuged for 10 min at 3000 rpm to separate the cellular and fluid phases. The plasma supernatant was collected and aliquoted. Samples were kept on ice until processing, processed fresh within one hour after collection and stored at -80 °C.

Complement component assays

Complement assays were two-antibody sandwich enzyme-linked immunosorbent assays (ELISA) with some exceptions. C1sC1inh uses 96-Well 1-Spot SECTOR plates from Meso Scale Discovery (MSD) with specific well volumes (Cat# L15Xa-3). C4a is a competitive ELISA with Biotin C4a and a single capture antibody. Black 96 well plates (Costar #3925) were coated with 75 μL of 1-10 ug/mL capture antibody in bicarbonate buffer (pH 9.4) overnight at 4℃. Next day, the plates were washed with dPBS (pH 7.4) and blocked with 3% bovine serum albumin (BSA) prepared in dPBS for 1 hour. Protein standards purified from human serum, purchased from Complement Tech were prepared in the range of 500-0.02 ng/mL in dPBS containing 0.3% BSA, 0.1% Tween-20, and 0.01M EDTA (assay buffer). CSF samples were diluted in the range of 10 to 5,000-fold in assay buffer. After removal of blocking buffer, standards and samples were added at 75 μL per well, incubated shaking at 300 rpm overnight at 4℃. Next day, the plates were then washed four times with dPBS containing 0.05% Tween-20 (wash buffer). Then, 75 μL of alkaline-phosphatase conjugated detection antibody was added to all wells. Plates were incubated for 1 hour shaking at room temperature, washed four times with wash buffer and developed using 75 μL of alkaline phosphatase substrate (Life Technologies, T2214). After incubation for 20 minutes, plates were read using a high sensitivity luminometer. Standards were fit using a four-parameter logistic fit and unknowns converted to concentration, corrected for dilution and then plotted.

**SUPPLEMENTARY TABLES**

Abbreviations

CSF: cerebrospinal fluid; LLOQ: lower limit of quantification; ULOQ: upper limit of quantification; AQP4+NMOSD: aquaporin-4-IgG seropositive neuromyelitis optica spectrum disorder; MOGAD: myelin oligodendrocyte glycoprotein antibody associated disease; MS: multiple sclerosis; NIC: non-inflammatory controls; HC: healthy controls; ON: optic neuritis; EDSS: expanded disability status scale

**Supplementary Table 1.** Quality control metrics for cytokine analytes

| Analyte | Number of samples below LLOQ | | Number of samples above ULOQ | | Number of samples with >30% difference between duplicates within the reported linear range | |
| --- | --- | --- | --- | --- | --- | --- |
|  | CSF | Plasma | CSF | Plasma | CSF | Plasma |
| IL-1a* | 115 | 130 | - | - | 1 | - |
| IL-1b* | 123 | 132 | - | - | 2 | 2 |
| IL-2* | 127 | 134 | - | - | - | - |
| IL-4* | 133 | 137 | - | - | - | 1 |
| IL-6 | 2 | - | 1 | - | 1 | - |
| IL-10 | 61 | 7 | - | - | - | 3 |
| IL-12 p70* | 126 | 111 | - | - | - | 2 |
| IL-17a | 20 | 64 | - | - | 6 | 17 |
| IFN-g* | 116 | 26 | - | - | - | 1 |
| TNF-a | 3 | - | - | - | 1 | - |
| BAFF | - | - | 1 | 8 | - | - |
| NfL | - | 9 | 6 | - | - | 5 |
| GFAP | - | 87 | 5 | - | 1 | - |

^Total number of samples: 135 CSF samples, 140 plasma samples

*CSF and plasma IL-1a, IL-1b, IL-2, IL-4, IL12 p70 and CSF IFN-g were not analyzed in downstream analyses due to >75% values missing or below the LLOQ

**Supplementary Table 2.** Associations of analytes with demographics (age, sex) in non-inflammatory controls in CSF and healthy controls in plasma

|  | **Non-inflammatory controls; CSF** | | **Healthy controls; plasma** | |
| --- | --- | --- | --- | --- |
| **Demographic feature** | **Age** | **Sex** | **Age** | **Sex** |
| IL-6 | r=0.02, p=0.92 | -0.21±0.25, p=0.41 | r=0.25, p=0.18 | -0.33±0.25, p=0.20 |
| IL-10 | r=0.04, p=0.79 | -0.13±0.12, p=0.27 | r=0.09, p=0.66 | -0.25±0.27, p=0.35 |
| IL-17a | **r=-0.30, p=0.042** | -0.10±0.13, p=0.45 | r=0.18, p=0.37 | 0.13±0.32, p=0.69 |
| IFN-g | - | - | r=0.10, p=0.61 | 0.33±0.52, p=0.53 |
| TNF-a | r=0.05, p=0.73 | -0.10±0.13, p=0.44 | r=-0.07, p=0.72 | 0.19±0.14, p=0.17 |
| BAFF | **r=0.38, p=0.008** | 0.03±0.10, p=0.74 | r=0.06, p=0.76 | -0.02±0.09, p=0.84 |
| NfL | **r=0.39, p=0.006** | 0.24±0.27, p=0.38 | **r=0.53, p=0.003** | 0.30±0.30, p=0.32 |
| GFAP | **r=0.43, p=0.002** | 0.27±0.22, p=0.23 | r=0.16, p=0.40 | 0.13±0.09, p=0.18 |
| Free C1q | **r=0.29, p=0.042** | -0.04±0.13, p=0.73 | - | - |
| C1s | **r=0.34, p=0.017** | 0.14±0.15, p=0.35 | - | - |
| C1inh | r=0.25, p=0.07 | 0.06±0.15, p=0.70 | - | - |
| C1s-C1inh | **r=0.37, p=0.008** | 0.04±0.17, p=0.82 | - | - |
| C2 | r=0.16, p=0.28 | -0.23±0.29, p=0.44 | - | - |
| C2b | r=0.27, p=0.054 | -0.29±0.36, p=0.42 | - | - |
| C3 | r=0.12, p=0.42 | 0.19±0.29, p=0.51 | - | - |
| C3a | r=0.15, p=0.31 | 0.20±0.29, p=0.50 | - | - |
| C3d | **r=0.32, p=0.023** | -0.03±0.23, p=0.90 | - | - |
| C4 | **r=0.39, p=0.005** | 0.14±0.15, p=0.36 | - | - |
| C4a | **r=0.32, p=0.023** | 0.15±0.23, p=0.51 | - | - |
| C5a | r=0.07, p=0.65 | -0.02±0.10, p=0.81 | - | - |
| C5b-9 | r=-0.004, p=0.98 | -0.06±0.37, p=0.87 | - | - |

Statistically significant results by Pearson’s correlation coefficient are highlighted in bold

**Supplementary Table 3.** Analyte concentrations and ranges in CSF

| **Group** | **AQP4+NMOSD** | **MOGAD** | **MS** | **Non inflammatory controls** |
| --- | --- | --- | --- | --- |
| **N** | **19** | **29** | **36** | **51** |
| IL-1a, median (range), pg/mL | 0.35 (0.35, 2.23) | 0.35 (0.35, 0.53) | 0.35 (0.35, 3.64) | 0.35 (0.35, 0.76) |
| IL-1b, median (range). pg/mL | 0.28 (0.28, 43.5) | 0.28 (0.28, 2.15) | 0.28 (0.28, 0.44) | 0.28 (0.28, 0.28) |
| IL-2, median (range), pg/mL | 0.38 (0.38, 0.68) | 0.38 (0.38, 0.56) | 0.38 (0.38, 0.7) | 0.38 (0.38, 0.54) |
| IL-4, median (range), pg/mL | 0.37 (0.37, 0.37) | 0.37 (0.37, 0.37) | 0.37 (0.37, 0.37) | 0.37 (0.37, 0.37) |
| IL-6, median (range), pg/mL | 3.42 (0.77, 2652) | 8.63 (0.79, 1987) | 2.48 (0.53, 19) | 1.95 (0.2, 26.8) |
| IL-10, median (range), pg/mL | 1.4 (0.41, 14.1) | 0.7 (0.41, 6.05) | 0.97 (0.41, 6.35) | 0.41 (0.41, 2.32) |
| IL-12 p70, median (range), pg/mL | 0.44 (0.44, 9.71) | 0.44 (0.44, 0.44) | 0.44 (0.44, 0.75) | 0.44 (0.44, 0.44) |
| IL-17a, median (range), pg/mL | 1.44 (0.74, 2.11) | 1.5 (0.74, 8.62) | 1.37 (0.74, 2.01) | 1.47 (0.74, 2.23) |
| IFN-g, median (range), pg/mL | 0.12 (0.12, 617) | 0.12 (0.12, 1.3) | 0.12 (0.12, 2.75) | 0.12 (0.12, 0.12) |
| TNF-a, median (range), pg/mL | 0.87 (0.21, 20.3) | 0.78 (0.3, 12.9) | 0.84 (0.36, 1.55) | 0.57 (0.21, 1.66) |
| BAFF, median (range), pg/mL | 131.5 (37.3, 840) | 134 (52.5, 633) | 71.5 (22.7, 124) | 112 (50.7, 197) |
| NfL, median (range), pg/mL | 2059.5 (196, 10290) | 1346 (118, 10290) | 2197.5 (174, 10290) | 486 (121, 9039) |
| GFAP, median (range), pg/mL | 245 (20.2, 9600) | 113 (5.29, 625) | 120 (25, 426) | 115 (24.1, 574) |
| Free C1q, median (range), ug/mL | 0.209 (0.085, 0.67) | 0.263 (0.087, 0.677) | 0.166 (0.061, 0.301) | 0.165 (0.088, 0.554) |
| C1s, median (range), ug/mL | 0.817 (0.227, 2.023) | 0.838 (0.244, 2.344) | 0.658 (0.102, 1.25) | 0.591 (0.157, 1.696) |
| C1inh, median (range), ug/mL | 1.102 (0.431, 3.717) | 1.386 (0.533, 4.132) | 0.883 (0.242, 2.143) | 0.907 (0.324, 3.314) |
| C1sC1inh, median (range), ug/mL | 0.022 (0.005, 1.527) | 0.013 (0.005, 0.102) | 0.01 (0.005, 0.19) | 0.007 (0.005, 0.043) |
| C2, median (range), ug/mL | 0.487 (0.045, 2.491) | 0.422 (0.077, 1.553) | 0.277 (0.022, 1.37) | 0.262 (0.018, 1.756) |
| C2b, median (range), ug/mL | 0.007 (0.003, 0.029) | 0.003 (0.001, 0.014) | 0.007 (0.001, 0.346) | 0.003 (0.001, 0.032) |
| C3, median (range), ug/mL | 6.803 (3.192, 31.331) | 9.252 (3.31, 70.083) | 3.306 (1.214, 31.775) | 4.71 (0.77, 25.963) |
| C3a, median (range), ug/mL | 0.1 (0.026, 0.318) | 0.126 (0.041, 0.417) | 0.064 (0.012, 0.278) | 0.051 (0.012, 0.234) |
| C3d, median (range), ug/mL | 1.864 (0.496, 4.108) | 1.652 (0.161, 8.493) | 1.207 (0.299, 3.841) | 0.844 (0.114, 4.795) |
| C4, median (range), ug/mL | 5.262 (0.415, 24.66) | 10.734 (1.262, 24.632) | 5.736 (0.774, 11.803) | 5.799 (1.952, 17.187) |
| C4a, median (range), ug/mL | 0.027 (0.006, 0.117) | 0.018 (0.003, 0.058) | 0.019 (0.002, 0.056) | 0.014 (0.002, 0.033) |
| C5a, median (range), ug/mL | 0.001 (0, 0.003) | 0.001 (0, 0.004) | 0.001 (0.001, 0.002) | 0.001 (0, 0.002) |
| C5b-9, median (range), ug/mL | 0.005 (0.005, 0.648) | 0.035 (0.005, 0.328) | 0.038 (0.005, 0.306) | 0.048 (0.005, 0.473) |

**Supplementary Table 4.** Analyte concentrations and ranges in plasma

| **Group** | **AQP4+NMOSD** | **MOGAD** | **MS** | **Healthy controls** |
| --- | --- | --- | --- | --- |
| **N** | **18** | **35** | **58** | **29** |
| IL-1a, median (range), pg/mL | 0.35 (0.35, 0.85) | 0.35 (0.35, 1.29) | 0.35 (0.35, 6.26) | 0.35 (0.35, 0.55) |
| IL-1b, median (range), pg/mL | 0.28 (0.28, 14.3) | 0.28 (0.28, 0.59) | 0.28 (0.28, 1.08) | 0.28 (0.28, 0.43) |
| IL-2, median (range), pg/mL | 0.38 (0.38, 0.7) | 0.38 (0.38, 0.38) | 0.38 (0.38, 0.7) | 0.38 (0.38, 2.92) |
| IL-4, median (range), pg/mL | 0.37 (0.37, 0.88) | 0.37 (0.37, 0.37) | 0.37 (0.37, 0.37) | 0.37 (0.37, 0.64) |
| IL-6, median (range), pg/mL | 2.47 (0.93, 14.6) | 2.05 (0.35, 20.2) | 1.88 (0.31, 67.8) | 2.28 (0.54, 5.67) |
| IL-10, median (range), pg/mL | 2.16 (0.65, 17.3) | 1.96 (0.41, 10.1) | 1.67 (0.41, 6.95) | 1.83 (0.41, 8.5) |
| IL-12 p70, median (range), pg/mL | 0.44 (0.44, 20.9) | 0.44 (0.44, 0.85) | 0.44 (0.44, 3.1) | 0.44 (0.44, 3.38) |
| IL-17a, median (range), pg/mL | 0.74 (0.74, 6.98) | 0.74 (0.74, 2.05) | 0.74 (0.74, 3.16) | 0.9 (0.74, 10.2) |
| IFN-g, median (range), pg/mL | 0.35 (0.12, 11.2) | 0.31 (0.12, 3.1) | 0.34 (0.12, 8.35) | 0.39 (0.12, 95.3) |
| TNF-a, median (range), pg/mL | 7.31 (4.21, 19.6) | 6.07 (2.48, 27.9) | 6.38 (2.12, 21.6) | 7.67 (2.42, 17.5) |
| BAFF, median (range), pg/mL | 458 (70.1, 840) | 417 (181, 840) | 455.5 (215, 840) | 476 (318, 811) |
| NfL, median (range), pg/mL | 24.7 (1.91, 188) | 19.95 (1.91, 696) | 19.6 (1.91, 182) | 7.6 (1.91, 24.4) |
| GFAP, median (range), pg/mL | 5.95 (1.78, 29.7) | 1.78 (1.78, 11.4) | 1.78 (1.78, 11.2) | 1.78 (1.78, 3.94) |

**Supplementary Table 5.** Comparison between groups for the CSF cohort

| **Comparison^**  **(beta ± SE)** | **NIC vs AQP4** | **NIC vs MOGAD** | **NIC vs MS** | **AQP4 vs MOGAD** | **AQP4 vs MS** | **MS vs MOGAD** |
| --- | --- | --- | --- | --- | --- | --- |
| IL-6 | **1.46±0.39, p<0.001**  (FDR: p<0.001) | **2.02±0.33, p<0.001**  (FDR: p<0.001) | 0.28±0.31, p=0.38  (FDR: p=0.46) | 0.56±0.42, p=0.19 | **-1.18±0.41, p=0.004** | **1.74±0.35, p<0.001** |
| IL-10 | **0.96±0.18, p<0.001**  (FDR: p<0.001) | **0.65±0.16, p<0.001**  (FDR: p<0.001) | **0.78±0.15, p<0.001**  (FDR: p<0.001) | -0.31±0.2, p=0.13 | -0.18±0.19, p=0.36 | -0.13±0.17, p=0.45 |
| IL-17a | 0.04±0.1, p=0.67  (FDR: p=0.71) | 0.16±0.09, p=0.06  (FDR: p=0.09) | -0.02±0.08, p=0.78  (FDR: p=0.82) | 0.12±0.11, p=0.28 | -0.07±0.1, p=0.53 | **0.18±0.09, p=0.046** |
| TNF-a | **0.65±0.17, p<0.001**  (FDR: p<0.001) | **0.6±0.15, p<0.001**  (FDR: p<0.001) | **0.36±0.14, p=0.01**  (FDR: p=0.018) | -0.05±0.19, p=0.8 | -0.29±0.18, p=0.11 | 0.24±0.16, p=0.13 |
| BAFF | 0.26±0.14, p=0.07  (FDR: p=0.11) | **0.41±0.12, p=0.001**  (FDR: p=0.003) | **-0.49±0.11, p<0.001**  (FDR: p<0.001) | 0.15±0.16, p=0.34 | **-0.74±0.15, p<0.001** | **0.89±0.13, p<0.001** |
| NfL | **1.08±0.28, p<0.001**  (FDR: p<0.001) | **1.1±0.23, p<0.001**  (FDR: p<0.001) | **1.32±0.22, p<0.001**  (FDR: p<0.001) | 0.01±0.3, p=0.97 | 0.24±0.29, p=0.41 | -0.23±0.25, p=0.36 |
| GFAP | **1.33±0.28, p<0.001**  (FDR: p<0.001) | 0.14±0.23, p=0.55  (FDR: p=0.61) | 0±0.21,  p=0.98  (FDR: p=0.98) | **-1.19±0.3, p<0.001** | **-1.34±0.29, p<0.001** | 0.14±0.24, p=0.56 |
| Free C1q | 0.17±0.12, p=0.16  (FDR: p=0.22) | **0.43±0.11, p<0.001**  (FDR: p<0.001) | -0.06±0.09, p=0.52  (FDR: p=0.60) | 0.25±0.14, p=0.07 | -0.23±0.13, p=0.07 | **0.49±0.12, p<0.001** |
| C1s | 0.22±0.13, p=0.11  (FDR: p=0.15) | **0.41±0.12, p=0.001**  (FDR: p=0.003) | 0.01±0.11, p=0.96  (FDR: p=0.97) | 0.19±0.16, p=0.23 | -0.21±0.14, p=0.13 | **0.4±0.13, p=0.003** |
| C1inh | 0.17±0.13, p=0.2  (FDR: p=0.27) | **0.4±0.12, p=0.001**  (FDR: p=0.003) | -0.06±0.1, p=0.55  (FDR: p=0.61) | 0.23±0.15, p=0.13 | -0.23±0.14, p=0.09 | **0.46±0.13, p<0.001** |
| C1s-C1inh | **1.27±0.23, p<0.001**  (FDR: p<0.001) | **0.58±0.21, p=0.006**  (FDR: p=0.012) | 0.33±0.18, p=0.06  (FDR: p=0.09) | **-0.69±0.26, p=0.009** | **-0.94±0.24, p<0.001** | 0.24±0.22, p=0.27 |
| C2 | **0.57±0.24, p=0.018**  (FDR: p=0.032) | 0.42±0.21, p=0.054  (FDR: p=0.09) | -0.03±0.18, p=0.85  (FDR: p=0.88) | -0.15±0.28, p=0.58 | **-0.6±0.25, p=0.016** | **0.45±0.23, p=0.048** |
| C2b | **0.71±0.3, p=0.02**  (FDR: p=0.038) | 0.12±0.27, p=0.65  (FDR: p=0.71) | **0.66±0.23, p=0.006**  (FDR: p=0.012) | -0.58±0.35, p=0.1 | -0.05±0.32, p=0.87 | -0.53±0.29, p=0.07 |
| C3 | 0.44±0.25, p=0.08  (FDR: p=0.11) | **0.83±0.22, p<0.001**  (FDR: p<0.001) | -0.13±0.19, p=0.51  (FDR: p=0.60) | 0.39±0.29, p=0.17 | **-0.56±0.26, p=0.03** | **0.96±0.24, p<0.001** |
| C3a | **0.6±0.23, p=0.01**  (FDR: p=0.018) | **0.88±0.2, p<0.001**  (FDR: p<0.001) | 0.21±0.17, p=0.23  (FDR: p=0.31) | 0.28±0.26, p=0.28 | -0.39±0.24, p=0.1 | **0.67±0.22, p=0.002** |
| C3d | **0.64±0.19, p<0.001**  (FDR: p=0.003) | **0.83±0.17, p<0.001**  (FDR: p<0.001) | **0.43±0.15, p=0.004**  (FDR: p=0.009) | 0.19±0.22, p=0.39 | -0.21±0.2, p=0.28 | **0.4±0.18, p=0.027** |
| C4 | -0.17±0.16, p=0.28  (FDR: p=0.36) | **0.58±0.15, p<0.001**  (FDR: p<0.001) | -0.1±0.12, p=0.42  (FDR: p=0.51) | **0.75±0.18, p<0.001** | 0.07±0.17, p=0.67 | **0.68±0.15, p<0.001** |
| C4a | **0.61±0.2, p=0.002**  (FDR: p=0.005) | 0.34±0.18, p=0.06  (FDR: p=0.09) | **0.44±0.15, p=0.005**  (FDR: p=0.012) | -0.28±0.23, p=0.23 | -0.18±0.2, p=0.38 | -0.1±0.19, p=0.6 |
| C5a | **0.42±0.12, p<0.001**  (FDR: p=0.001) | **0.32±0.1, p=0.003**  (FDR: p=0.006) | **0.27±0.09, p=0.003**  (FDR: p=0.008) | -0.1±0.13, p=0.44 | -0.16±0.12, p=0.2 | 0.05±0.11, p=0.64 |
| C5b-9 | **-1.03±0.38, p=0.008**  (FDR: p=0.015) | **-0.72±0.34, p=0.038**  (FDR: p=0.06) | -0.26±0.29, p=0.38  (FDR: p=0.46) | 0.31±0.44, p=0.49 | 0.77±0.4, p=0.06 | -0.46±0.36, p=0.21 |

^Group listed first represents the reference group for each comparison

Statistically significant results are highlighted in bold. Benjamini-Hochberg false discovery rate (FDR)-adjusted p-values are shown in parentheses.

**Supplementary Table 6.** Comparison between groups for the plasma cohort

| **Comparison^**  **(beta ± SE)** | **HC vs AQP4** | **HC vs MOGAD** | **HC vs MS** | **AQP4 vs MOGAD** | **AQP4 vs MS** | **MS vs MOGAD** |
| --- | --- | --- | --- | --- | --- | --- |
| IL-6 | 0.21±0.27, p=0.44  (FDR: p=0.60) | 0.15±0.22, p=0.5  (FDR: p=0.60) | 0.2±0.2, p=0.32  (FDR: p=0.52) | -0.06±0.27, p=0.84 | -0.01±0.25, p=0.97 | -0.05±0.19, p=0.8 |
| IL-10 | 0.37±0.22, p=0.1  (FDR: p=0.34) | 0.19±0.18, p=0.31  (FDR: p=0.52) | -0.07±0.16, p=0.68  (FDR: p=0.68) | -0.19±0.23, p=0.42 | **-0.44±0.21, p=0.04** | 0.25±0.15, p=0.1 |
| IL-17a | -0.11±0.17, p=0.49  (FDR: p=0.60) | -0.21±0.15, p=0.16  (FDR: p=0.43) | -0.23±0.13, p=0.07  (FDR: p=0.28) | -0.09±0.17, p=0.59 | -0.12±0.15, p=0.46 | 0.02±0.12, p=0.86 |
| IFN-g | -0.16±0.32, p=0.62  (FDR: p=0.65) | -0.23±0.26, p=0.4  (FDR: p=0.59) | -0.14±0.24, p=0.55  (FDR: p=0.60) | -0.07±0.32, p=0.83 | 0.02±0.3, p=0.96 | -0.08±0.22, p=0.71 |
| TNF-a | -0.07±0.12, p=0.55  (FDR: p=0.60) | -0.15±0.1, p=0.13  (FDR: p=0.38) | -0.1±0.09, p=0.27  (FDR: p=0.52) | -0.08±0.12, p=0.51 | -0.03±0.11, p=0.81 | -0.05±0.08, p=0.52 |
| BAFF | -0.11±0.11, p=0.31  (FDR: p=0.52) | -0.11±0.09, p=0.22  (FDR: p=0.49) | -0.06±0.08, p=0.47  (FDR: p=0.60) | 0±0.11, p>0.99 | 0.05±0.1, p=0.61 | -0.05±0.07, p=0.49 |
| NfL | **1.07±0.3, p<0.001**  (FDR: p=0.003) | **0.99±0.25, p<0.001**  (FDR: p<0.001) | **1.04±0.22, p<0.001**  (FDR: p<0.001) | -0.09±0.31, p=0.78 | -0.04±0.29, p=0.9 | -0.05±0.21, p=0.81 |
| GFAP | **1.11±0.15, p<0.001**  (FDR: p<0.001) | 0.16±0.13, p=0.22  (FDR: p=0.49) | **0.25±0.11, p=0.03**  (FDR: p=0.14) | **-0.95±0.16, p<0.001** | **-0.86±0.14, p<0.001** | -0.09±0.11, p=0.39 |

^Group listed first represents the reference group for each comparison

Statistically significant results are highlighted in bold. Benjamini-Hochberg false discovery rate (FDR)-adjusted p-values are shown in parentheses.

**Supplementary Table 7.** Correlations between CSF and plasma levels of cytokines and neuroglial proteins

| **Analyte** | **Correlation between CSF and plasma levels** |
| --- | --- |
| IL-6 | r=0.26, p=0.17 |
| IL-10 | r=0.01, p=0.98 |
| IL-17a | r=0.18, p=0.40 |
| TNF-a | r=0.28, p=0.14 |
| **BAFF** | **r=0.51, p=0.005** |
| **NfL** | **r=0.79, p<0.001** |
| **GFAP** | **r=0.60, p<0.001** |

Statistically significant results are highlighted in bold

**Supplementary Table 8.** Correlations of CSF cytokines and complement proteins with CSF neuroglial proteins in AQP4+NMOSD, MOGAD and MS

|  | AQP4+NMOSD | | MOGAD | | MS | |
| --- | --- | --- | --- | --- | --- | --- |
|  | NfL | GFAP | NfL | GFAP | NfL | GFAP |
| IL-6 | r=0.27, p=0.29 | **r=0.86, p<0.001** | r=0.09, p=0.66 | r=0.20, p=0.30 | r=-0.08, p=0.64 | r=0.16, p=0.37 |
| IL-10 | r=0.36, p=0.15 | **r=0.85, p<0.001** | r=0.17, p=0.39 | r=0.31, p=0.11 | r=-0.15, p=0.39 | r=0.01, p=0.96 |
| IL-17a | r=-0.10, p=0.70 | r=0.12, p=0.66 | r=-0.16, p=0.42 | r=-0.19, p=0.34 | r=-0.23, p=0.20 | r=-0.25, p=0.16 |
| TNF-a | **r=0.58, p=0.014** | **r=0.69, p=0.003** | r=0.11, p=0.59 | **r=0.39, p=0.042** | r=-0.07, p=0.68 | **r=0.43, p=0.011** |
| BAFF | r=0.31, p=0.23 | **r=0.69, p=0.003** | r=0.17, p=0.40 | r=0.37, p=0.053 | **r=0.41, p=0.013** | r=0.25, p=0.15 |
| Free C1q | r=0.07, p=0.81 | **r=0.61, p=0.015** | **r=0.45, p=0.036** | **r=0.55, p=0.008** | r=-0.04, p=0.83 | r=0.23, p=0.18 |
| C1s | r=-0.15, p=0.58 | r=0.41, p=0.13 | r=0.37, p=0.09 | **r=0.49, p=0.021** | r=0.01, p=0.96 | r=0.07, p=0.70 |
| C1inh | r=0.23, p=0.40 | r=0.41, p=0.13 | **r=0.54, p=0.009** | **r=0.58, p=0.005** | r=0.04, p=0.8 | r=0.17, p=0.34 |
| C1s-C1inh | r=0.21, p=0.44 | **r=0.86, p<0.001** | **r=0.46, p=0.03** | **r=0.44, p=0.039** | r=-0.11, p=0.55 | r=-0.23, p=0.19 |
| C2 | r=0.11, p=0.71 | r=-0.28, p=0.34 | **r=0.71, p<0.001** | **r=0.69, p<0.001** | r=0.09, p=0.62 | **r=0.38, p=0.026** |
| C2b | r=0.41, p=0.13 | r=0.27, p=0.35 | **r=0.72, p<0.001** | **r=0.73, p<0.001** | r=-0.13, p=0.45 | r=0.18, p=0.29 |
| C3 | r=0.26, p=0.35 | r=0.16, p=0.59 | r=0.42, p=0.052 | r=0.39, p=0.07 | r=0.11, p=0.53 | r=0.07, p=0.69 |
| C3a | r=0.25, p=0.36 | r=0.28, p=0.33 | r=0.39, p=0.07 | r=0.35, p=0.11 | r=0.01, p=0.93 | r=0.08, p=0.65 |
| C3d | r=0.26, p=0.35 | r=0.36, p=0.20 | **r=0.56, p=0.007** | **r=0.64, p=0.001** | r=0.18, p=0.31 | r=0.31, p=0.07 |
| C4 | r=-0.16, p=0.54 | r=0.23, p=0.41 | **r=0.50, p=0.017** | **r=0.69,** **p<0.001** | r=0.14, p=0.43 | r=0.26, p=0.14 |
| C4a | r=0.35, p=0.18 | **r=0.70, p=0.004** | **r=0.60, p=0.003** | **r=0.68, p<0.001** | **r=0.34, p=0.045** | r=0.28, p=0.10 |
| C5a | r=-0.01, p=0.98 | r=-0.40, p=0.15 | r=0.21, p=0.35 | r=0.26, p=0.24 | r=-0.23, p=0.19 | r=-0.07, p=0.70 |
| C5b-9 | r=0.47, p=0.07 | r=0.21, p=0.47 | **r=0.56, p=0.006** | r=0.39, p=0.07 | r=-0.02, p=0.90 | r=-0.04, p=0.80 |

Statistically significant results are highlighted in bold

**Supplementary Table 9.** Associations of CSF analytes with clinical characteristics in AQP4+NMOSD

|  | Comparison between participants with ON (n=4; reference group) vs non-ON (n=15) attacks | Comparison between participants with samples collected within 60 days of attack symptom onset (n=13) vs 60-180 days (n=6; reference group) | Correlation of CSF analytes with EDSS at nadir (n=15) | Comparison between participants without (n=9; reference group) vs with exposure to steroids within 60 days (n=10) |
| --- | --- | --- | --- | --- |
| IL-6 | 1.19±1.13, p=0.3 | 0.58±1.01, p=0.57 | rho=0.49, p=0.07 | -1.09±0.91, p=0.25 |
| IL-10 | 0.56±0.49, p=0.27 | 0.27±0.45, p=0.55 | rho=0.38, p=0.16 | **-0.94±0.35, p=0.016** |
| IL-17a | 0.03±0.18, p=0.87 | -0.06±0.15, p=0.70 | rho=-0.09, p=0.75 | 0.06±0.13, p=0.65 |
| TNF-a | 0.70±0.52, p=0.19 | -0.21±0.48, p=0.66 | rho=0.41, p=0.12 | -0.68±0.42, p=0.12 |
| BAFF | 0.65±0.46, p=0.18 | 0.08±0.41, p=0.84 | **rho=0.56, p=0.033** | -0.29±0.36, p=0.43 |
| NfL | **2.10±0.71, p=0.01** | -0.95±0.70, p=0.19 | rho=0.37, p=0.18 | -0.63±0.65, p=0.34 |
| GFAP | **2.55±1.20, p=0.05** | 0.54±1.13, p=0.64 | **rho=0.52, p=0.05** | -1.29±1.00, p=0.22 |
| Free C1q | 0.18±0.40, p=0.66 | 0.27±0.31, p=0.39 | **rho=0.58, p=0.032** | 0.06±0.30, p=0.84 |
| C1s | 0.12±0.40, p=0.76 | 0.37±0.30, p=0.24 | **rho=0.54, p=0.047** | 0.18±0.29, p=0.56 |
| C1inh | 0.27±0.40, p=0.51 | 0.35±0.31, p=0.27 | rho=0.41, p=0.15 | -0.06±0.30, p=0.85 |
| C1s-C1inh | 1.01±0.91, p=0.29 | 0.64±0.73, p=0.39 | **rho=0.64, p=0.017** | -0.93±0.66, p=0.18 |
| C2 | -0.02±0.67, p=0.98 | 0.40±0.44, p=0.38 | rho=0.30, p=0.32 | 0.16±0.43, p=0.71 |
| C2b | -0.24±0.44, p=0.60 | 0.20±0.30, p=0.51 | rho=0.32, p=0.29 | -0.12±0.29, p=0.68 |
| C3 | 0.20±0.59, p=0.73 | 0.02±0.40, p=0.97 | rho=-0.03, p=0.93 | -0.15±0.38, p=0.70 |
| C3a | 0.14±0.50, p=0.79 | 0.04±0.34, p=0.92 | rho=-0.04, p=0.89 | -0.30±0.32, p=0.36 |
| C3d | 0.60±0.50, p=0.25 | -0.02±0.36, p=0.95 | rho=0.10, p=0.74 | 0.07±0.34, p=0.84 |
| C4 | 0.08±0.57, p=0.89 | 0.72±0.42, p=0.10 | rho=0.29, p=0.31 | 0.07±0.43, p=0.87 |
| C4a | 0.45±0.60, p=0.46 | -0.05±0.48, p=0.92 | **rho=0.59, p=0.027** | **-0.96±0.39, p=0.024** |
| C5a | -0.37±0.47, p=0.44 | 0.12±0.32, p=0.72 | rho=0.17, p=0.57 | 0.18±0.30, p=0.57 |
| C5b-9 | 1.36±1.15, p=0.26 | 1.44±0.72, p=0.06 | rho=0.08, p=0.78 | -0.21±0.77, p=0.79 |

Statistically significant results are highlighted in bold

**Supplementary Table 10.** Associations of CSF analytes with clinical characteristics in MOGAD

|  | Comparison between participants with ON (n=20; reference group) vs non-ON (n=9) attacks | Correlation of CSF analytes with EDSS at nadir (n=25) | Correlation of CSF analytes with follow-up EDSS if no interval relapse (n=17) | Comparison between participants without (n=14; reference group) vs with exposure to steroids within 60 days (n=15) |
| --- | --- | --- | --- | --- |
| IL-6 | **2.47±0.79, p=0.004** | rho=0.30, p=0.14 | rho=0.38, p=0.13 | -0.97±0.83, p=0.25 |
| IL-10 | **1.09±0.28, p<0.001** | **rho=0.45, p=0.024** | rho=0.48, p=0.053 | -0.55±0.30, p=0.08 |
| IL-17a | 0.28±0.20, p=0.17 | rho=0.27, p=0.20 | rho=0.39, p=0.14 | -0.16±0.19, p=0.40 |
| TNF-a | **1.30±0.31, p<0.001** | **rho=0.45, p=0.027** | rho=0.29, p=0.25 | -0.54±0.34, p=0.13 |
| BAFF | **0.87±0.18, p<0.001** | rho=0.25, p=0.22 | rho=0.19, p=0.46 | -0.11±0.23, p=0.63 |
| NfL | 0.42±0.44, p=0.35 | rho=0.21, p=0.30 | rho=0.28, p=0.27 | 0.76±0.39, p=0.06 |
| GFAP | 0.65±0.38, p=0.10 | rho=0.28, p=0.17 | rho=0.03, p=0.89 | 0.49±0.36, p=0.18 |
| Free C1q | **0.63±0.24, p=0.015** | **rho=0.45, p=0.05** | rho=0.46, p=0.10 | 0.36±0.23, p=0.13 |
| C1s | 0.48±0.25 p=0.06 | rho=0.38, p=0.09 | rho=0.29, p=0.31 | 0.33±0.22, p=0.15 |
| C1inh | **0.50±0.23, p=0.04** | **rho=0.47, p=0.039** | rho=0.42, p=0.14 | 0.24±0.21, p=0.27 |
| C1s-C1inh | **0.86±0.29, p=0.008** | rho=0.38, p=0.09 | rho=0.50, p=0.07 | 0.31±0.30, p=0.32 |
| C2 | 0.52±0.37, p=0.17 | rho=0.37, p=0.11 | **rho=0.55, p=0.045** | -0.23±0.33, p=0.50 |
| C2b | **0.93±0.39, p=0.025** | rho=0.34, p=0.15 | rho=0.32, p=0.26 | -0.41±0.37, p=0.28 |
| C3 | 0.63±0.39, p=0.12 | rho=0.41, p=0.07 | rho=0.52, p=0.058 | 0.61±0.34, p=0.09 |
| C3a | 0.50±0.28, p=0.09 | rho=0.44, p=0.055 | rho=0.52, p=0.06 | **0.52±0.24, p=0.044** |
| C3d | 0.55±0.36, p=0.14 | rho=0.41, p=0.07 | rho=0.42, p=0.13 | 0.56±0.31, p=0.08 |
| C4 | 0.39±0.32, p=0.24 | rho=0.03, p=0.90 | rho=0.03, p=0.92 | 0.32±0.28, p=0.28 |
| C4a | **0.77±0.30, p=0.017** | **rho=0.48, p=0.033** | rho=0.42, p=0.13 | 0.28±0.30, p=0.35 |
| C5a | -0.10±0.27, p=0.71 | rho=-0.32, p=0.17 | rho=-0.19, p=0.51 | -0.15±0.23, p=0.51 |
| C5b-9 | 0.45±0.76, p=0.56 | rho=0.04, p=0.85 | rho=-0.02, p=0.93 | 1.19±0.62, p=0.07 |

Statistically significant results are highlighted in bold

**Supplementary Table 11.** Associations of CSF analytes with clinical characteristics in MS

|  | Comparison between participants with samples collected within 60 days of attack symptom onset (n=25) vs 60-180 days (n=11; reference group) | Correlation of CSF analytes with EDSS at nadir (n=29) | Correlation of CSF analytes with follow-up EDSS if no interval relapse (n=23) | Comparison between participants without (n=20; reference group) vs with exposure to steroids within 60 days (n=16) |
| --- | --- | --- | --- | --- |
| IL-6 | -0.21±0.27, p=0.44 | rho=0.08, p=0.69 | rho=-0.12, p=0.57 | -0.27±0.25, p=0.28 |
| IL-10 | **0.53±0.25, p=0.044** | rho=0.26, p=0.18 | rho=0.14, p=0.52 | -0.12±0.25, p=0.62 |
| IL-17a | 0.03±0.11, p=0.75 | rho=-0.36, p=0.07 | rho=-0.40, p=0.07 | -0.02±0.10, p=0.81 |
| TNF-a | -0.05±0.12, p=0.66 | rho=0.05, p=0.8 | rho=0.31, p=0.14 | -0.09±0.11, p=0.43 |
| BAFF | **-0.36±0.18, p=0.05** | rho=0.01, p=0.97 | rho=0.04, p=0.85 | -0.25±0.17, p=0.14 |
| NfL | 0.13±0.38, p=0.74 | rho=0.14, p=0.46 | rho=0.18, p=0.41 | 0.30±0.35, p=0.39 |
| GFAP | -0.18±0.25, p=0.48 | rho=-0.10, p=0.6 | rho=0.12, p=0.59 | -0.07±0.23, p=0.75 |
| Free C1q | 0.11±0.11, p=0.34 | rho=0.04, p=0.82 | rho=0.28, p=0.20 | -0.04±0.10, p=0.70 |
| C1s | 0.18±0.17, p=0.30 | rho=-0.07, p=0.72 | rho=0.21, p=0.34 | 0.14±0.16, p=0.39 |
| C1inh | 0.13±0.14, p=0.36 | rho=-0.22, p=0.25 | rho=0.11, p=0.60 | 0.06±0.13, p=0.68 |
| C1s-C1inh | 0.07±0.29, p=0.81 | rho=0.02, p=0.92 | rho=-0.18, p=0.40 | -0.04±0.27, p=0.88 |
| C2 | 0.39±0.27, p=0.16 | rho=-0.28, p=0.14 | rho=-0.02, p=0.93 | -0.04±0.26, p=0.87 |
| C2b | -0.12±0.47, p=0.8 | rho=-0.26, p=0.17 | rho=0.002, p=0.99 | -0.39±0.43, p=0.37 |
| C3 | 0.33±0.34, p=0.34 | rho=-0.30, p=0.12 | rho=0.04, p=0.84 | -0.11±0.32, p=0.73 |
| C3a | 0.37±0.29, p=0.21 | rho=-0.22, p=0.24 | rho=-0.02, p=0.94 | -0.15±0.28, p=0.58 |
| C3d | 0.16±0.21, p=0.44 | rho=-0.09, p=0.65 | rho=0.01, p=0.97 | -0.22±0.19, p=0.27 |
| C4 | 0.13±0.18, p=0.46 | rho=-0.17, p=0.37 | rho=0.14, p=0.54 | 0.02±0.17, p=0.93 |
| C4a | 0.19±0.23, p=0.41 | rho=-0.10, p=0.59 | rho=0.22, p=0.31 | 0.13±0.22, p=0.55 |
| C5a | 0.05±0.12, p=0.69 | rho=0.06, p=0.76 | rho=-0.14, p=0.51 | -0.08±0.11, p=0.43 |
| C5b-9 | 0.25±0.49, p=0.61 | rho=-0.10, p=0.60 | rho=-0.06, p=0.80 | -0.03±0.45, p=0.94 |

Statistically significant results are highlighted in bold
